# Further investigation of phenotypes and confounding factors of progressive ratio performance and feeding behavior in the BACHD rat model of Huntington disease

**DOI:** 10.1371/journal.pone.0173232

**Published:** 2017-03-08

**Authors:** Erik Karl Håkan Clemensson, Laura Emily Clemensson, Benedikt Fabry, Olaf Riess, Huu Phuc Nguyen

**Affiliations:** 1 Institute of Medical Genetics and Applied Genomics, Tuebingen, Tuebingen, Germany; 2 Centre for Rare Diseases, Tuebingen, Tuebingen, Germany; 3 QPS Austria, Grambach, Austria; Northeastern Ohio Medical University, UNITED STATES

## Abstract

Huntington disease is an inherited neurodegenerative disorder characterized by motor, cognitive, psychiatric and metabolic symptoms. We recently published a study describing that the BACHD rat model of HD shows an obesity phenotype, which might affect their motivation to perform food-based behavioral tests. Further, we argued that using a food restriction protocol based on matching BACHD and wild type rats’ food consumption rates might resolve these motivational differences. In the current study, we followed up on these ideas in a longitudinal study of the rats’ performance in a progressive ratio test. We also investigated the phenotype of reduced food consumption rate, which is typically seen in food-restricted BACHD rats, in greater detail. In line with our previous study, the BACHD rats were less motivated to perform the progressive ratio test compared to their wild type littermates, although the phenotype was no longer present when the rats’ food consumption rates had been matched. However, video analysis of food consumption tests suggested that the reduced consumption rate found in the BACHD rats was not entirely based on differences in hunger, but likely involved motoric impairments. Thus, restriction protocols based on food consumption rates are not appropriate when working with BACHD rats. As an alternative, we suggest that studies where BACHD rats are used should investigate how the readouts of interest are affected by motivational differences, and use appropriate control tests to avoid misleading results. In addition, we show that BACHD rats display distinct behavioral changes in their progressive ratio performance, which might be indicative of striatal dysfunction.

## Introduction

Huntington disease (HD) is an autosomal dominantly inherited neurodegenerative disorder, which is caused by a specific mutation in the gene for the huntingtin protein [1,2]. The mutation concerns an expansion of the CAG repeat sequence present in the gene’s first exon, which results in an elongated stretch of glutamine in the translated protein. Patients who carry an allele with more than 40 CAG repeats invariably develop HD [3,4]. During the disease process there is extensive neuronal loss, starting in the caudate nucleus of the striatum, but eventually encompassing most brain regions [5–7]. This results in a wide range of clinical signs that are commonly grouped into motor, psychiatric, cognitive and metabolic symptoms [8]. There are currently no disease-modifying treatments available for HD, and the disease is invariably fatal [2,8,9].

Several different transgenic animal models of HD have been generated [2, 10–14]. Thus, a large amount of work in HD research concerns the characterization of these animal models to better understand which aspects of the disease are well represented in a given model, which ones are not present, and which aspects might be unique to the model itself. When considering behavioral characterization studies, one also has to consider that as the models are likely to show a range of different phenotypes (disease-related or not), some might confound the readouts of others. As an example, metabolic phenotypes have been found to confound tests that assess motoric function [15,16].

Our group primarily works with the BACHD rat model of HD. These rats carry a transgenic construct that contains the full-length disease-causing human gene with 97 CAG/CAA repeats [17]. We recently published a study where we concluded that male BACHD rats, similar to other HD models that carry the full-length disease-causing gene, show a strong obesity phenotype [18]. Interestingly, we found that although the rats were obese, their body weight was still similar to that of their wild type (WT) littermates due to developmental deficits (reduced body size, disproportionally low muscle weight). In addition, the obesity phenotype persisted despite the fact that the BACHD rats generally consumed less food compared to WT rats [18].

One of the reasons for us favoring a rat model over any of the mouse models was the wider range of cognitive tests that are available for rats. However, the apparent metabolic phenotypes of the male BACHD rats raised some concerns. Specifically, we were concerned that these phenotypes might result in BACHD rats being less motivated than WT rats when performing various tests of cognitive function, as many of these are based on working for food rewards [19]. Motivational differences have been shown to affect both apparent cognitive abilities and choice of strategy in the Barnes maze [20]. For most cognitive tests, it is not known how a motivational difference affects the animals’ performance. Thus, interpretations of behavioral phenotypes found in an animal model that might show reduced motivation should be done carefully.

In our initial study we therefore ran a progressive ratio test to assess male BACHD rats’ motivation to perform lever pushes for a food reward [18]. Specifically, we assessed the performance during both a standard and an alternative food restriction protocol. The standard food restriction protocol was based on common practice, where all animals are food restricted until they reach a specified body weight, typically 85% of their free-feeding weight [18,19]. Using this protocol, we found that BACHD rats were less motivated than their WT littermates to perform the test. This was an interesting phenotype on its own, as it might be related to apathy symptoms that are frequently found in HD patients [21,22]. However, as the BACHD rats are obese without showing an increased body weight it would also mean that they likely carried more adipose tissue compared to WT rats during this restriction protocol. This would in turn mean that they likely had an increased serum concentration of leptin, a protein that is secreted from adipose tissue and regulates energy metabolism [23]. Importantly, changes in leptin signaling within the central nervous system have been shown to affect motivation in the progressive ratio test [24–26]. Specifically, increased leptin levels are able to reduce motivation [24,25], while knock-down of leptin receptors can increase motivation [26]. Thus, the reduced motivation among male BACHD rats might have been a result of their metabolic phenotypes. The alternative food restriction protocol aimed to elucidate this. Rather than being based on reaching a specific relative body weight, this protocol was based on adjusting the rats’ food restriction level so that their apparent hunger and food interest was similar [18]. The rats’ apparent hunger was assessed by measuring their food consumption rates in a test where they were given free access to food during 15 minutes. When maintained on the standard food restriction protocol, male BACHD rats consumed food at a lower rate compared to WT rats, although this could be resolved by giving WT rats an increased daily amount of food. When BACHD and WT rats showed comparable food consumption rates, there was no longer any difference in motivation to perform the progressive ratio test. Thus, we suggested that motivational differences between BACHD and WT rats can be expected when using standard food restriction protocols, that these phenotypes are likely caused by metabolic phenotypes rather than psychiatric phenotypes, and that the alternative food restriction protocol might be more suitable to use during tests of cognitive characterization [18].

The study itself still had certain shortcomings, which we have sought to cover in the follow-up study presented here. Briefly, our first study only considered rats of relatively young ages (2–4 months of age) and we here aimed to further investigate to what extent the findings were reproduced at older ages. Further, we have investigated the rats’ body composition during the alternative food restriction protocols as well as how the leptin levels among BACHD and WT rats changed during different parts of our tests (i.e. during the different food restriction protocols). Additional control tests have been performed in order to exclude fatigue and satiation as confounding factors in the progressive ratio results. Finally, more detailed evaluation of the food consumption test used for assessing the rats’ apparent hunger, and a separate test allowing assessment of individual animals’ feeding behavior, have been performed in order to better understand the nature of the reduced food consumption rate seen among male BACHD rats.

## Material and methods

### Animals

A total of 48 male rats were used for the study. These were acquired from two separate in-house breeding events, with hemizygous BACHD males from the TG5 line [17] paired with WT females (Crl:CD(SD), Charles River, Germany). All animals were on Sprague-Dawley background. Animals were genotyped according to previously published protocols [17] and housed in genotype-matched groups of three in type IV cages (38 × 55cm), with high lids (24.5cm from cage floor). Rats had free access to water through the entire study. During experiments, body weight was measured daily to track the rats’ relative food restriction level and assess basic health. Between experiments, body weight was measured weekly. During experiments, rats were food restricted according to two protocols described in detail below and in [18]. During both protocols, each cage was given a specific daily amount of food (SNIFF V1534-000 standard chow) to maintain appropriate restriction levels. Rats had free access to food between the experiments.

The animal facility kept 21–23°C, 55–10% humidity, and was set to a partially inverted light/dark cycle with lights on/off at 02:00/14:00 during summer, and 01:00/13:00 during winter.

**Fig 1:**
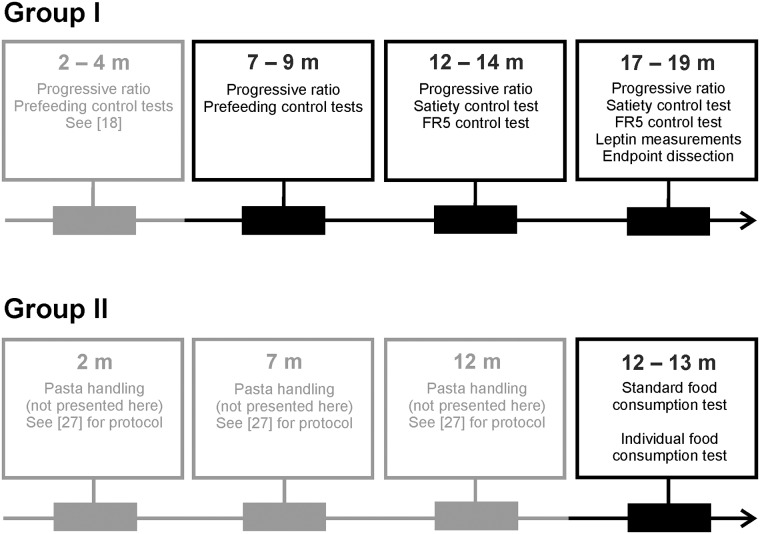
Study overview. The study used two groups of rats that were assessed in different behavioral tests, as indicated in the figure. The horizontal arrows indicate the time frame during which the work was performed, with the different tests ages indicated in text boxes. Gray-colored boxes and text indicate tests that are presented elsewhere, but constitute important information about the rats’ behavioral testing experience. Group I was used in a longitudinal progressive ratio test with a total of four test ages. Different control tests were used at different ages, as detailed in the Material and Methods section. The results from the first age are presented elsewhere [18]. Group II was used for the detailed analysis of the reduced food consumption rate seen among BACHD rats. This analysis was only performed at a single test age. The group had previous experience in a pasta-handling test, the results of which will be published elsewhere.

The 48 rats were split into two groups of 24 rats, both composed of 12 WT and 12 BACHD rats. The first group was used for a longitudinal progressive ratio test, leptin measurements and endpoint dissection to investigate body composition. This group will be referred to as Group I. The second group was used for a longitudinal pasta-handling test, although the results from this are not considered here (unpublished data) (see [27] for protocol). They were also used for the detailed study of BACHD rats’ food consumption phenotypes, which is presented here. This group will be referred to as Group II. Group I was tested at 2, 7, 12 and 17 months of age in the progressive ratio test, while the leptin measurements were only performed at the last age. The results from the test at 2 months were presented in our previous publication [18] and will only be referred to in this publication. Group II was assessed in the pasta-handling test at 2, 7 and 12 months of age. The detailed study of BACHD rats’ food consumption presented here was performed at the end of their 12 months experiment. Fig 1 presents an overview of the tests performed with the two different animal groups.

All experiments were approved by the local ethics committee (Regierungspraesidium Tuebingen) and carried out in accordance with the German Animal Welfare Act and the guidelines of the Federation of European Laboratory Animal Science Associations, based on European Union legislation (Directive 2010/63/EU).

### Food restriction protocols

As noted above, two different food restriction protocols were used throughout the study. The first one focused on restricting the animals to a specific relative body weight. During this, both BACHD and WT rats were restricted until they reached 85% of their respective free-feeding body weight. This relative body weight, or food restriction level, was calculated using previously gathered data from growth curves of BACHD and WT rats. Thus, the calculations could be made with gender, age and genotype-matched values and took normal growth into account. This protocol was used as the start point for all tests described below, and will be referred to as the standard food restriction protocol.

Once data from performance on the standard food restriction protocol had been gathered, the restriction was changed to the alternative protocol. As noted above, this restriction was based on the rats’ food consumption rates (assessed in a test described in [18] and below), rather than their relative body weight. During this, the amount of food given to the WT rats was increased, while the amount given to BACHD rats was kept more or less constant, until WT and BACHD rats showed similar food consumption rates. At that point, data for a second baseline was gathered.

It should be noted that it was rarely possible to give the exact same amount of food during extended periods of time to either of the genotypes, as both the standard and alternative restriction still had to take natural growth into account.

### Progressive ratio

As mentioned above, Group I was used for a longitudinal experiment using the progressive ratio test. This was the same group of animals that had been used for our initial study [18], and only the results from their test runs at 7, 12 and 17 months of age will be presented here. A detailed description of the protocol and setup is available elsewhere [18], and is only described briefly in the current publication.

Behavioral assessment started 30 minutes after dark phase onset, in a room separate from the animals’ housing room, using soft red light. A bank of six operant conditioning chambers (Coulbourn Instruments, H10-11R-TC) was used to run the test. Each chamber was equipped with two retractable levers, one on either side of a central pellet receptacle trough equipped with a yellow light. This light was used to signal the delivery of a reward pellet. The chambers contained a red house light on the wall opposite from the levers and pellet receptacle trough, which shone during the full duration of the training sessions. A water bottle was also available on this wall, to ensure *ad libitum* access to water during testing. The progressive ratio protocol was designed and run with Graphic State 4.1.04. Rats were given single daily sessions, meaning that a total of four daily runs with all six operant chambers were needed to assess the whole group. Each run assessed three WT and three BACHD rats in a determined order, so that a given rat was trained on the same time of day through all tests. Each rat was assigned to a specific operant chamber, although this was arranged so that each operant chamber was used to assess equal numbers of WT and BACHD rats. Rats received their daily amount of regular food four hours after the completion of the last run of the day.

At each test age the rats were first put on food restriction for approximately 14 days. This aimed at restricting both WT and BACHD rats to 85% of their respective free-feeding body weights, as described above. At the first test age, all rats were then habituated to the operant conditioning boxes and subjected to initial lever-training protocols before finally being trained on the progressive ratio protocol. These steps are described in detail elsewhere [18]. For all subsequent ages (i.e. the results presented in the current publication), rats were directly trained on the final progressive ratio protocol, as no other retraining appeared to be necessary.

The main aim of the progressive ratio test is to assess how many lever pushes a rat is willing to perform in order to get a reward pellet (Bio-serv, Dustless Precision Pellets^®^ F0021, purchased through Bilaney consultants, Duesseldorf, Germany). At the start of each test session, both levers were extended into the conditioning chamber, allowing rats to interact with them. The levers remained in this position for the full duration of the test session. One lever was reinforced, while the other one was non-reinforced. The exact position (i.e. left or right lever) of the reinforced and non-reinforced lever was counter-balanced for the two genotypes and remained constant for individual rats through all experiments. Pushing the reinforced lever resulted in reward pellets being delivered. At the start of each session, the rats needed to push five times in order to receive a reward pellet. After ten completed ratios, i.e. ten pellets received, the number of required pushes increased after each completed ratio. The increase was made in an arithmetic fashion within each block of ten ratios, but also changed between the blocks, to give an overall exponential progression. Thus, during the first, second and third block of ten ratios, the ratio requirement increased with one, three and five pushes per completed ratio, respectively. The sessions lasted 80 minutes. The main behavioral parameter of interest was a set of break points, defined as the first ratio where a rat made no responses on the reinforced lever during 10, 25, 50, 100, 300 or 600 seconds. Rats were trained until both genotype groups had reached a stable performance. A baseline was then constructed from the last few sessions as detailed below.

Once a baseline had been achieved using the standard food restriction protocol, the alternative food restriction protocol was initiated. During this, the rats were still given daily progressive ratio sessions, but in addition, a food consumption test was run each day at the time when the rats would normally receive their daily amount of food. As noted above, WT rats were then given an increased amount of food until they showed a comparable food consumption rate to BACHD rats. At that point, data for a stable baseline of progressive ratio performance was once again gathered. When a second baseline had been obtained, the rats were put back on free feeding and the test ended.

Although the exact number of sessions used for the different progressive ratio baselines presented in this publication differed, none used fewer than six consecutive sessions. It should also be noted that the feeding test was run on a weekly basis during training on the standard food restriction protocol. As mentioned in [18], the training took a substantial amount of time at each age, and despite the intention of assessing the rats’ behavior at 2, 7, 12 and 17 months of age, the more exact ages for the baselines presented in [18] and here are 2–4, 7–9, 12–14 and 17–19 months of age.

Several parameters were analyzed in addition to the set of break points described above. These included the total number of completed ratios (i.e. rewards obtained), the total number of pushes performed on the reinforced lever, the total number of pushes performed on the non-reinforced lever and several parameters regarding the latency to retrieve the reward pellets. For this, there was first the full retrieval latency, calculated from the delivery of the pellet to the point where the rat entered the pellet trough to retrieve it. This parameter was then split. This produced the latency to leave the reinforced lever, which measured the time from delivery of the reward pellet to the OFF-signal of the last lever push the rat performed on the reinforced lever. The latency to move from the lever to the pellet trough was then calculated separately, measuring the time from the OFF-signal of the last lever push to the point when the rat entered the pellet trough. Two additional parameters were added to describe the latency to leave the reinforced lever in greater detail. The first one calculated the number of excessive pushes (i.e. additional pushes performed after the delivery of the reward pellet) that the rats performed on the reinforced lever before retrieving the pellet. The result of this parameter was expressed as the mean number of excessive pushes performed per completed ratio. The other parameter calculated the latency to leave the lever specifically on ratios where no excessive pushes were performed, and was called the latency to release the reinforced lever.

Separate analysis for the first ten FR5 ratios was also performed, including a set of slightly different parameters. These constituted the latency to perform the first lever push, the time needed to complete a given ratio, the latency to return to the reinforced lever after retrieving the reward pellet and the pellet retrieval latency (calculated as the full retrieval latency explained above).

### Progressive ratio control tests

In our initial study [18], a set of prefeeding tests was used to further evaluate the motivational difference between WT and BACHD rats. On each test occasion, the rats were fed a fixed amount of either regular food or reward pellets prior to performing the progressive ratio test. The resulting drop in motivation was then analyzed and discussed. In total, the rats were assessed in four different test sessions, which were presented on alternating days with normal progressive ratio tests. These prefeeding tests were repeated at the 7–9 months test age. However, on that occasion both WT and BACHD rats failed to return to their baseline performance during sessions that separated the prefeeding tests. Instead, the rats gradually became less motivated with each prefeeding test being run. Because of this, the results were excluded from the current manuscript. In addition, the prefeeding tests were not rerun at the subsequent test ages.

During the 12–14 and 17–19 months test ages, the rats’ progressive ratio performance was also assessed at satiety, before food restriction according to the standard protocol was initiated. We hypothesized that the results would be similar to the ones obtained when using the alternative food restriction protocol, as WT and BACHD rats should in both cases be equally hungry and/or satiated. These tests used the same basic progressive ratio protocol, but the sessions were only 45 minutes long. In addition, the test sessions were started two hours after the dark-phase onset, to give both WT and BACHD rats ample time to finish their main feeding bout of the dark phase.

Another control test was added during the 12–14 and 17–19 months test ages. In this protocol, there was no progression, and the required number of lever pushes was kept at five pushes through the entire session (FR5 protocol). Single sessions of this protocol were run after establishing the satiety baseline at 12–14 months, and all three baselines at 17–19 months of age (i.e. satiety, standard food restriction and alternative food restriction). The sessions were run on the same time schedule as the standard progressive ratio protocol, had the same maximum duration, but sessions also ended once a rat had acquired 200 pellets. This protocol was run in order to investigate if the motivational differences in progressive ratio performance might have been caused by BACHD rats becoming fatigued or satiated during the sessions.

### Leptin measurements

During the 17–19 months test age of Group I, blood samples were collected after establishing each progressive ratio baseline (i.e satiety, standard food restriction and alternative food restriction). At each stage, the blood samples were collected the day after the FR5 control test had been run. In addition, a fourth set of blood samples was collected at the endpoint of the experiment, when rats were sacrificed and dissected as described below. Samples were collected during the same time of day on all occasions. The first three sets of samples were collected from the rats’ tail vein. This was done by inserting a needle of 0.6 mm diameter into the vein and collecting roughly 1 ml of whole blood into a microcentrifuge tube. No anesthesia or specific fixation method were required for this procedure, as the rats had been extensively handled by the experimenters during the study. After collection, the samples were allowed to clot while being kept on ice, and were then centrifuged at 5°C with 1000*g*, for 30 minutes. The resulting blood serum was collected and stored at -80°C until ELISA analysis was performed approximately 10 months later.

Leptin concentrations were measured at QPS Austria GmbH (Grambach, Austria) using a Quantikine ELISA kit (Mouse/Rat leptin Quantikine ELISA kit, R&D systems, Austria, Vienna). Serum samples from animals at satiety were diluted 1:10 and 1:20 for WT and BACHD rats, respectively. For all other samples, dilution series of 1:2.5, 1:5 and 1:10 were prepared. The final sample preparation resulted in an additional 1:2 dilution, according to the kit’s accompanying protocol. Concentration measurement was based on the supplied leptin standard. Duplicate samples were analyzed for satiety samples. For other samples, a mean concentration was calculated based on 1–3 samples, depending on how many samples from the dilution series were within the range of the standard curve. For most samples, this resulted in duplicate measurements.

### Body composition analysis

After completing the set of tests run at 17–19 months of age, the rats of Group I were sacrificed while they were still maintained on the alternative food restriction protocol. Briefly, the rats were sacrificed in a carbon dioxide chamber two to four hours before dark-phase onset. Body lengths and body weights were then measured on the intact animals, with body length measured from nose tip to tail tip. Additional measurements of head, trunk and tail length were taken from nose tip to back of the head, back of the head to anus and anus to tail tip, respectively. Afterwards, blood samples were collected transcardially and processed as described above. The rats were then subjected to a detailed dissection aimed at investigating their body composition. First, skin and subcutaneous adipose tissue deposits were removed and weighed. Then, internal organs and adipose deposits located in the abdomen and chest cavity were removed and weighed. The remaining carcass was weighed to obtain a measurement of bone and muscle weight (denoted bone/muscle). The dissection of Group I was performed during four consecutive days.

### Standard food consumption test

The standard food consumption test was used at several points during the study to assess the rats’ food consumption rates and formed the basis of the alternative food restriction protocol. The protocol for this test has been described in our initial study of the BACHD rats’ food consumption rates [18], and similar protocols have been described by others [28–33]. The aim of the test is to acquire a basic measurement of the rats’ apparent interest in food, i.e. hunger levels. For this, a small amount of food was placed in the cage tops of the rats’ homecages (approximately 50 g, the exact weight differed between cages (+/- 5 g), but was carefully noted, down to two decimals). The food was then left there for 15 minutes. Afterwards, the remaining food in each cage was measured.

As noted above, the food consumption tests were run in connection to the actual time of feeding for the rats. After calculating how much food the rats consumed during the test, this amount was subtracted from the cages’ daily food amount.

For Group I, this test was run weekly during the progressive ratio training when rats were maintained on the standard food restriction protocol, and daily during the progressive ratio training when rats were maintained on the alternative food restriction protocol. For Group II, where characterizing the food consumption rate phenotype was the primary aim, the test was run daily during both food restriction protocols. Specifically, the rats’ behavior during the standard food restriction protocol was first assessed during eight consecutive days to establish a baseline of their performance. Afterwards, they were run in the individual food consumption test as described below. Once that had been completed, the rats were run on the standard food consumption test for an additional three sessions. During these three days, videos of the rats’ performance were recorded. Afterwards, a single session was run where the food was placed on the cage floors instead of the cage tops. When all of that was done, the rats were put on the alternative food restriction, and the standard food consumption test was once again run daily, until BACHD and WT rats showed similar food consumption rates. At that point, the rats were again run in the individual food consumption test. After this, the rats were assessed in the standard food consumption test during three consecutive days in order to gather videos of their performance. The video scoring of the tests is described in detail below.

### Individual food consumption test

The fact that the standard food consumption test is run in groups, leads to some drawbacks. As an example, detailed scoring of the number of bites and duration of chewing episodes cannot reliably be scored from videos of the test. Because of this, we also sought to evaluate the consumption rates and feeding behavior of individual animals, in Group II. Through their pasta-handling test (data not shown), the rats had been extensively habituated to a roughly cube-shaped glass cage (28.5 × 29 × 29.5 cm, also described in [18]). Because of this, they readily consumed regular food inside the same setup, which made them suitable for the current study. In addition, the setup allowed for good quality close-up videos of the rats’ behavior.

As noted above, the rats were assessed in this test after stable baselines of their performance in the standard food consumption test had been established (during both food restriction protocols). Each animal was given single daily sessions where they were placed inside the glass cage and given a single food piece. The trial then continued until the rats had consumed the food piece. The entire trial was video-recorded to allow for subsequent video scoring (see below). The food pieces had been filed down to approximately 2.4 g (+/- 0.1 g) (the exact weight of each food piece was noted, down to two decimals) to achieve consistent weight and blunt edges for all trials. During both the standard and alternative food restriction, several sessions were run in order to establish stable baseline performance. At the end of the test, the rats’ head length, from nose tip to the back of the head, was measured.

### Video analysis

As noted, video recordings of both the standard and the individual food consumption tests were made to better investigate the nature of the phenotypes that had been found. During scoring, experimenters were blinded to the rats’ genotypes, while this was not the case when the videos were gathered. All video scoring was performed using the Observer XT software (v.12.5.927, Noldus, The Netherlands, Wageningen). The following behaviors were scored for the standard food consumption test:

#### Food-oriented behaviors

This included all behaviors that could be argued to be food-oriented. In addition to the more specific behaviors noted below, this primarily considered occasions when the rats appeared to be searching through the bedding material for food pieces, but in general included most behaviors performed at or around the food crib. In contrast, behaviors where the rats investigated smells and sounds from outside the cage, or general activity in the part of the cage that was not situated below the food crib, was not considered food-oriented.

#### Food crib attention

Episodes of food-crib attention were scored when the rats clearly investigated the food inside the food crib. Naturally, this included the time they actively spent biting on food pieces, but also occasions where they only sniffed the food or clearly angled their heads towards it while being in its direct vicinity.

#### Biting episode

This was specifically scored when the rats where actively biting or trying to bite the food pieces in the food crib.

#### Consuming a separate food piece

On occasion, rats would bite off a larger food piece, or find a food piece in the bedding material below the food crib. They would then frequently take the piece in their mouth, walk away from the food crib and sit still in another part of the cage. Although it was rarely directly visible, it was assumed that they were then actively consuming the food piece, which was scored as a separate behavior. The behavior was clearly distinguishable from both grooming and resting, as the rats sat very still in a hunched position, rather than performing typical grooming movements or lying down.

Through the tests sessions, these behaviors occurred in episodes of different durations. For each behavior, the total number of episodes, the mean episode duration and the total time spent doing a specific type of behavior was calculated. From this, the total time spent on two other behavioral parameters were calculated. General food crib attention was calculated by subtracting the total time of biting episodes from the total time spent paying attention to the food crib. The parameter thus described the total time the rats spent on more cursory investigations of the food crib. Other food-oriented behaviors was calculated by subtracting the total time spent paying attention to the food crib and the total time spent consuming a separate food piece from the total time spent on arguably food-oriented behaviors. Finally, the latency to initiate biting was calculated for food crib attention episodes where biting occurred.

For both the standard and alternative food restriction protocols, only one video per cage was analyzed. The videos were chosen so that the rats’ food consumption rate on the analyzed session was a good approximation of their baseline performance. For a given cage, scoring was made on each individual rat, although the tail and ear markings that were used for identifying them were not visible on the videos. Thus, the rats were given arbitrary names based on their position inside the cage at the session start, to keep them apart during scoring.

The scoring of the individual food consumption test focused on the detailed behavior of how the rats consumed single food pieces. In general, the rats spent essentially no time doing general exploration of the setup, so a separate scoring of this was not necessary. Thus, the following parameters were scored:

#### Time needed to consume the food piece

Rats were considered to be feeding when clearly biting and gnawing on the food piece. In addition, making clear chewing motions when either holding the food piece or standing in its direct vicinity and remaining focused on it was considered active feeding. Rats were not considered to be actively feeding if they were walking around investigating the setup or were clearly not focusing on the food pellet, even if these behaviors often included some chewing motions. In addition, eating food dust from the cage floor was excluded from the active feeding time. Still, it should be noted that these behaviors were rare.

#### Number, duration and frequency distribution of biting episodes

A biting episode was considered any phase where the rats were actively biting or gnawing pieces off of the main food piece. The start of these episodes was clearly identifiable with the rat using its forepaws to lift the food piece upwards, and simultaneously lowering its head, in order to position the food piece into its open mouth. The specific nature of the biting episode could then be quite varying, although the rat typically either bit a single piece off or performed several gnawing motions with its lower jaw. The end of the biting episode, and the start of the chewing episode, was then scored when the rat lifted its head from the food piece and started chewing. In addition to calculating the total number and mean duration of biting episodes, the frequency distribution of biting episodes with different durations was analyzed. This analysis used 15 bins of 0.2 seconds, and a final bin containing biting episodes that were longer than three second.

#### Number, duration and frequency distribution of chewing episodes

Once the rat had managed to bite a piece off from the main food piece, it typically spent some time chewing before returning to bite another piece off. The chewing episodes were considered to end when the rat initiated another biting episode. Through this, the bouts of active feeding were split into several alternating biting and chewing episodes. In addition to calculating the total number and mean duration of chewing episodes, the frequency distribution of chewing episodes of different durations was analyzed. This analysis used 25 bins of 0.2 seconds, and five bins of three seconds for longer chewing episodes.

On some occasions the rats bit off pieces that were too large to eat in a single bite. The rats would then drop the main food piece and hold on to the piece that was bitten off, in order to bite smaller pieces off from it. These events were scored as a single biting episode, as no chewing was initiated. On other occasions, the rats would bite a piece off and then spend some time using small mouth movements to get the whole piece into their mouths before actually starting to chew it. On these occasions, the chewing episode was considered to start from the point that the rats had bitten the piece off in order to include also the small mouth movements. Thus, the biting episodes included behaviors that aimed at getting a comfortable food piece off of the food pellet while the chewing episodes included behaviors that focused on managing to chew and swallow those food pieces.

In addition to the parameters above, the theoretical bite size for each rat was calculated based on the number of biting episodes the rats had made and the measured weight of the food pellet. Further, the food consumption rate was calculated based on the food pellet’s weight and the time needed to consume it.

### Statistical analysis

Analysis of baseline performance during the progressive ratio test comprised of several different graphing and analysis methods. Single comparisons of BACHD and WT performance were subjected to *t*-test, *t*-test with Welch correction or Mann-Whitney test depending on the data’s apparent distribution. Parameters presented in curves were analyzed with two-way repeated measures ANOVAs using in most cases genotype as between-subject factor and break point, age, food restriction protocol or behavioral protocol as within-subject factor. Sidak’s multiple comparison *post-hoc* test was used to follow up on any significant effects of genotype, or on interaction effects found in the two-way ANOVAs. Analysis of performance during the FR5 part of the progressive ratio protocol (i.e. performance during the first ten ratios) was performed in the same manner, but with ratio being used as within-subject factor. During the study, some rats became ill and had to be sacrificed. Thus the n of the analyses changed as follows: 7–9 months data (WT: 12, BACHD: 11), 12–14 months data (WT: 12, BACHD: 11) and 17–19 months data (WT: 12, BACHD: 9 for data from standard food restriction, WT: 11, BACHD: 9 for data from alternative food restriction). Analysis of age progression excluded animals for which data was not available at all ages. No other exclusion criteria were used.

To gain further information of the rats’ progressive ratio performance, data from the final break point (break point 600) from all baselines established during standard and alternative food restriction was analyzed in a three-way ANOVA. The analysis used genotype as between-subject factor and age and food restriction protocol as within-subject factors. Significant two-way interactions were graphed and pairwise analyses were made using Sidak’s multiple comparison *post-hoc* test. As the analysis included age, data from rats that had been sacrificed before the end of the study were excluded. This put the n for the analysis at 11 for WT and 9 for BACHD rats.

Parameters investigated in connection to leptin level analysis were analyzed through a series of single comparisons between BACHD and WT rats, using *t*-test, *t*-test with Welch correction or Mann-Whitney test depending on the data’s apparent distribution. Curves and ANOVAs were avoided due to the strong non-normal distribution in WT rats’ leptin levels, which was found to influence statistical readouts and obscure the findings concerning the alternative food restriction protocol. The current approach was chosen to avoid excluding experimentally sound data. Analysis was performed on the 11 WT and 9 BACHD rats for which progressive ratio data and blood samples were available at all three baselines (satiety, standard food restriction and alternative food restriction). WT rats were, in addition, subjected to paired analysis of body weight, leptin levels and BP600 for the two different food restriction protocols.

Parameters from dissection results were also analyzed in a series of single comparisons between BACHD and WT rats, using *t*-test, *t*-test with Welch correction or Mann-Whitney test depending on the data’s apparent distribution.

Curves comparing mean baseline food consumption rates during standard and alternative food restriction protocols, for both the standard and individual food consumption tests, were analyzed with two-way repeated measures ANOVA. As above, these used genotype as between-subject factor and restriction protocol as within-subject factor. Sidak’s multiple comparison *post-hoc* test was used to follow up on any significant effects of genotype, or interaction effects. In addition, performance of WT rats was subjected to paired analysis, comparing the performance on both restriction settings. Additional curves showing food consumption rate on all test sessions are included in the figures for descriptive purpose. The standard food consumption test was based on mean consumption rates for cages, resulting in an n of 4 for both WT and BACHD rats. The individual food consumption test was based on individual performances. Group II consisted of a total of 12 WT and 12 BACHD rats. However, 2 WT rats had to be excluded from the analysis, as they did not reliably consume the food piece during the alternative food restriction protocol, leaving an n of 10 WT and 12 BACHD rats.

The video analysis of the standard food consumption test focused on a series of individual comparisons between WT and BACHD rat performance, using *t*-test, *t*-test with Welch correction or Mann-Whitney test depending on the data’s apparent distribution. No specific analysis of behavioral changes due to the change of food restriction protocol was performed, although additional graphs depicting the change, but using the statistics of the individual comparisons, were made. This was because the rats’ actual identity was not visible in the videos, and thus repeated measures analysis could not be performed. Scoring within each baseline performance was done on an individual basis, giving an n of 12 for both WT and BACHD rats.

Video analysis of behavior during the individual food consumption test was only performed for the alternative food restriction protocol, as the restriction protocol did not appear to have any effect on food consumption rate in this test. Analysis consisted of a series of individual comparisons between WT and BACHD rat performance, using *t*-test, *t*-test with Welch correction or Mann-Whitney test depending on the data’s apparent distribution. In addition, the distribution of biting and chewing episodes of different durations were analyzed with two-way repeated measures ANOVA using genotype as between-subject factor and episode duration as within-subject factor. The analysis was performed on both absolute numbers of episodes and data related to the total number of episodes performed. No *post-hoc* analysis was performed. As noted above, the analysis used 10 WT and 12 BACHD rats. An additional distribution analysis with fewer episode duration bins was also performed. This analysis used a series of individual comparisons between BACHD and WT rats, applying tests describe above, rather than a two-way ANOVA.

Alpha for all analyses was set to 0.05. The three-way ANOVA was performed with SPSS statistics v.20.0.0 (IBM Corporation, Armonk, New York, USA, http://www.ibm.com). All other statistical analyses were conducted using GraphPad Prism v.6.01 (GraphPad Software, San Diego California USA, http://www.graphpad.com).

## Results

### Survival

Most rats remained healthy through the entire duration of the study, and only a few rats (three BACHD and one WT rat from Group I) were sacrificed due to illness. In all cases, the illnesses concerned tumors. Although the higher incidence of sacrifice among BACHD rats in this study might suggest that BACHD rats show a generally shorter life span than WT rats, we have not seen any consistent indications of this when considering all studies performed at our institute.

### Progressive ratio

**Fig 2:**
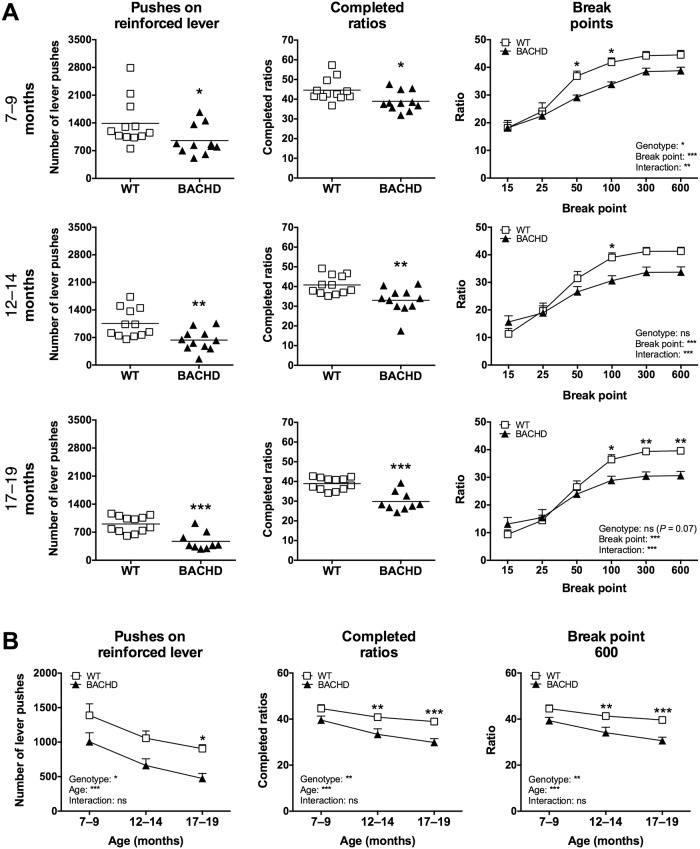
Primary readouts of progressive ratio performance during standard food restriction. The graphs show the performance of Group I in the progressive ratio test, when rats were maintained on the standard food restriction protocol. (A) displays the baseline performance at the three older ages. The mean number of pushes performed on the reinforced lever and mean number of completed ratios are displayed in scatter plots, where each data point represents an individual animal's performance. The groups' mean values are also indicated. The graphs for break point analysis display the ratio where a given break point was reached, with group mean plus standard error being shown. (B) displays the age progression of the main readouts. The graphs indicate group mean plus standard error. For the scatter plots, significant results from *t*-test or Mann-Whitney test are shown inside the graphs. For (B) as well as for all break point graphs, repeated two-way ANOVA results are displayed inside the graphs, and results from *post-hoc* analysis are shown for individual data points in case significant genotype differences were detected. (*P* < 0.05) *, (*P* < 0.01) ** and (*P* < 0.001) ***.

**Fig 3:**
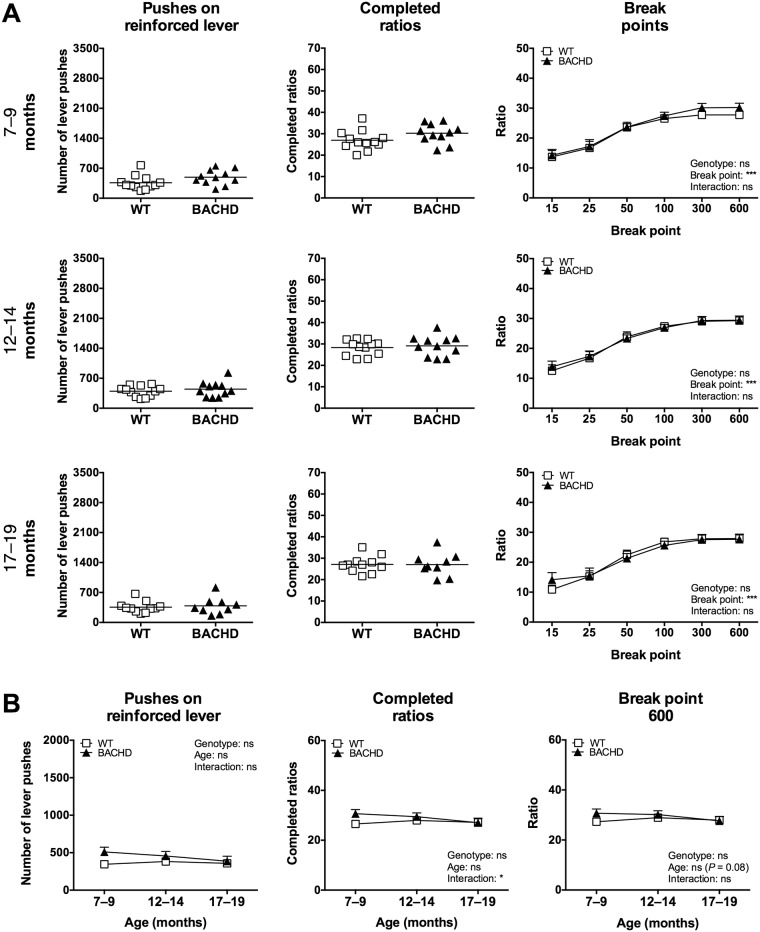
Primary readouts of progressive ratio performance during alternative food restriction. The graphs show the performance of Group I in the progressive ratio test, when animals were food restricted so that their food consumption rates were matched. (A) displays the baseline performance at the three older ages. The mean number of pushes performed on the reinforced lever and mean number of completed ratios are displayed in scatter plots, where each data point represents an individual animal's performance. The groups' mean values are also indicated. The graphs for break point analysis display the ratio where a given break point was reached, with group mean plus standard error being shown. (B) displays the age progression of the main readouts. The graphs indicate group mean plus standard error. For the scatter plots, significant results from *t*-test or Mann-Whitney test are shown inside the graphs. For (B) as well as for all break point graphs, repeated two-way ANOVA results are displayed inside the graphs, and results from *post-hoc* analysis are shown for individual data points in case significant genotype differences were detected. (*P* < 0.05) *, (*P* < 0.01) ** and (*P* < 0.001) ***.

The results from Group I’s performance on the progressive ratio test at four months of age [18] were well reproduced when the rats were retested at older ages in the current study (Figs 2 and 3). Specifically, BACHD rats performed fewer pushes on the reinforced lever, completed fewer ratios and reached lower breakpoints compared to WT rats when the standard food restriction protocol was used (Fig 2A). Rats of both genotypes appeared to be gradually less motivated to perform the test as they aged (Fig 2B), although the motivational differences between the genotypes remained largely unchanged. Still, *post-hoc* analysis revealed that a subtle progression effect might be present. When using the alternative food restriction protocol, the genotype differences were no longer present and BACHD and WT rats consistently showed similar levels of motivation in the progressive ratio test (Fig 3A). This was primarily due to a clear drop in motivation among WT rats, although performance also dropped slightly among BACHD rats. Performance on the alternative food restriction protocol showed no statistically significant change with age, although weak trends indicated that the motivation dropped slightly (Fig 3B). Pushes on the non-reinforced lever were rare for both genotypes at all ages and on both food restriction protocols, with no indication of genotype or interaction effects (S1 Fig). Rats of both genotypes performed their highest number of non-reinforced lever pushes during the 7–9 months test period when the standard food restriction protocol was used. At all following baselines, the number of non-reinforced pushes appeared to remain stable.

**Fig 4:**
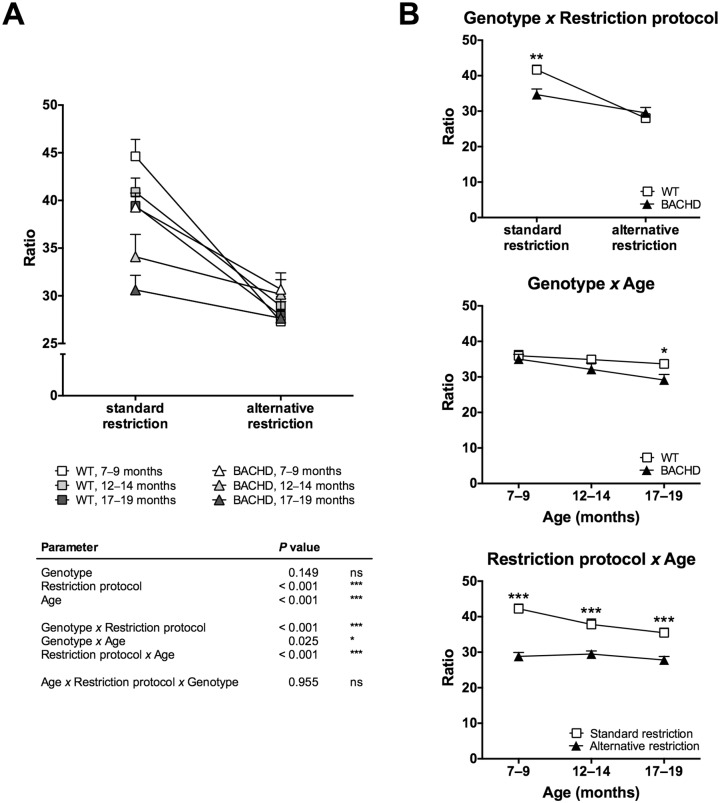
Three-way ANOVA analysis of break point 600. The graphs show the results from a three-way ANOVA analysis of break point 600 for the performance baselines displayed in Figs 2 and 3. (A) displays all included data points and a summary table of the statistics. (B) displays plots for the significant two-way interaction effects. All graphs display group mean plus standard error. In (B), results from pairwise comparisons with Sidak’s multiple comparison *post-hoc* test are displayed for data points that differed significantly from each other. (*P* < 0.05) *, (*P* < 0.01) ** and (*P* < 0.001) ***.

The results from the three-way ANOVA analysis of break point 600 supported the results described above and added certain analysis elements (Fig 4). The ANOVA did not reveal any overall effect of genotype, while both the restriction protocol and age had a general impact on break point 600 (Fig 4A). Further, each of the reported two-way interactions (Genotype x Restriction protocol, Genotype x Age, and Restriction protocol x Age) were significant, although the Genotype x Age interaction was considerably weaker than the others (Fig 4A). The three-way interaction (Restriction protocol x Age x Genotype) was, in contrast, not significant (Fig 4A). The significant two-way interactions were subjected to further analysis (Fig 4B). From this, it was once again noted that although both WT and BACHD rats dropped in motivation between the two baselines, the effect was stronger among WT rats. This effect likely caused the significant Genotype x Restriction protocol interaction. The analysis further indicated that as rats grew older, their performance appeared to drop at a faster rate among BACHD rats compared to WT rats. This likely caused the significant Genotype x Age interaction effect. Finally, the performance difference between rats maintained on the standard and alternative food restriction protocols was particularly strong during the 7–9 months test age. This likely caused the significant Restriction protocol x Age interaction effect.

**Fig 5:**
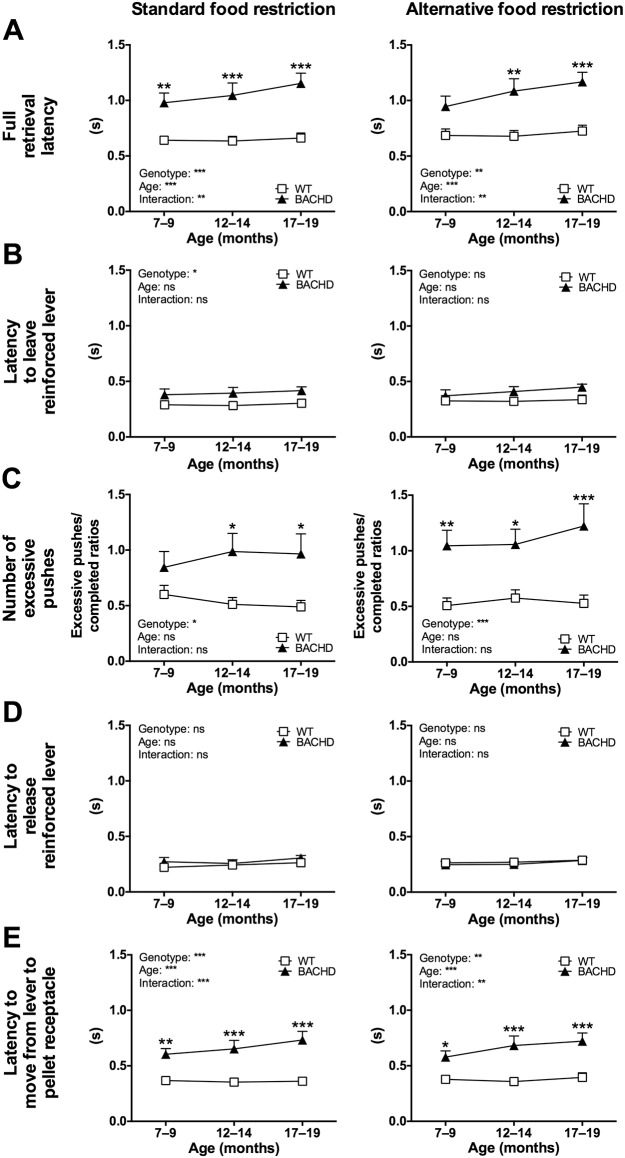
Detailed analysis of pellet retrieval latency during the progressive ratio test. The graphs show age progression of various parameters related to the latency to retrieve the reward pellet during progressive ratio testing of Group I. Results from both food restriction protocols are shown. (A) shows the full retrieval latency, while (B)–(E) show its individual components. Detailed information on how the different parameters were measured is described in the Material and Methods section. The graphs indicate group mean plus standard error. Repeated two-way ANOVA results are displayed inside each graph, and results from *post-hoc* analysis are shown for individual data points in case significant genotype differences were detected. (*P* < 0.05) *, (*P* < 0.01) ** and (*P* < 0.001) ***.

BACHD rats were consistently found to have longer full pellet retrieval latencies compared to WT rats, regardless of which food restriction protocol was used (Fig 5A). As described in the Material and Methods section, the full pellet retrieval latency was composed of the latency to leave the reinforced lever and the time needed to move from the reinforced lever to the pellet receptacle. BACHD rats were slightly slower than WT rats in terms of leaving the reinforced lever (Fig 5B), which appeared to be caused by them making a higher number of excessive lever pushes before retrieving the pellet (Fig 5C), rather than having problems with simply releasing the lever (Fig 5D). In addition, BACHD rats were consistently found to be slower than WT rats in moving from the reinforced lever to the pellet trough (Fig 5E), which likely represented the main cause of their slowed full retrieval latency. Concerning age progression, WT rats showed stable pellet retrieval latencies, while BACHD rats appeared to become slower as they were retested (Fig 5A and 5E). The number of excessive lever pushes (Fig 5C), and other parameters (Fig 5B and 5D), remained arguably stable with increasing age.

There were no striking differences between the BACHD and WT rats’ performance during the FR5 phase of the progressive ratio test (S2 Fig). Still, there was a trend indicating that BACHD rats needed longer time than WT rats to complete the very first ratio of the session (S2B Fig). In addition, BACHD rats were again found to need significantly longer time than WT rats to retrieve the reward pellets on both food restriction protocols (S2C Fig).

### Progressive ratio control tests

**Fig 6:**
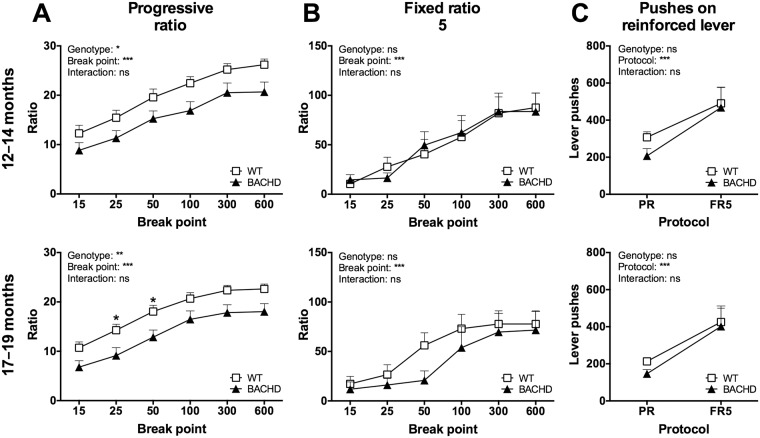
Progressive ratio and FR5 control test performance during satiety. The graphs show performance of Group I in the progressive ratio and FR5 control tests when rats were maintained on free-feeding conditions. (A) shows break point analyses for progressive ratio testing at 12–14 and 17–19 months of age. (B) shows break point analyses for FR5 testing at the same ages. (C) shows comparisons of the mean number of lever pushes performed on the reinforced lever during the two test protocols. The graphs display group mean plus standard error. Repeated two-way ANOVA results are displayed above each graph, and results from *post-hoc* analysis are shown for individual data points in case significant genotype differences were detected. (*P* < 0.05) *, (*P* < 0.01) ** and (*P* < 0.001) ***.

During the test performed at 2–4 months of age, we used a prefeeding control test [18]. The aim was to control for differences in the BACHD and WT rats’ hunger levels. As mentioned in the Material and Methods section, this was repeated for the 7–9 months test, but the results were excluded, as the rats did not reliably return to their baselines between the prefeeding tests. A separate set of control tests was thus added at 12–14 and 17–19 months of age. On both occasions, the rats were assessed in the progressive ratio test and in an FR5 test at satiety. During the 17–19 months test, the FR5 protocol was also run after establishing the progressive ratio baselines for the standard and alternative food restriction protocols. At satiety, BACHD rats were less motivated than WT rats to perform the progressive ratio test (Fig 6A), but were equally motivated to perform the FR5 test (Fig 6B). This was true for both test ages. Importantly, both BACHD and WT rats completed more ratios (Fig 6A and 6B) and performed more pushes on the reinforced lever (Fig 6C) during the FR5 protocol compared to the progressive ratio protocol. When comparing progressive ratio test performances during satiety and the standard food restriction protocol, rats of both genotypes showed increased motivation to lever-push for rewards on the latter. This effect appeared to be somewhat stronger among WT rats, particularly at the last test age (S3 Fig).

**Fig 7:**
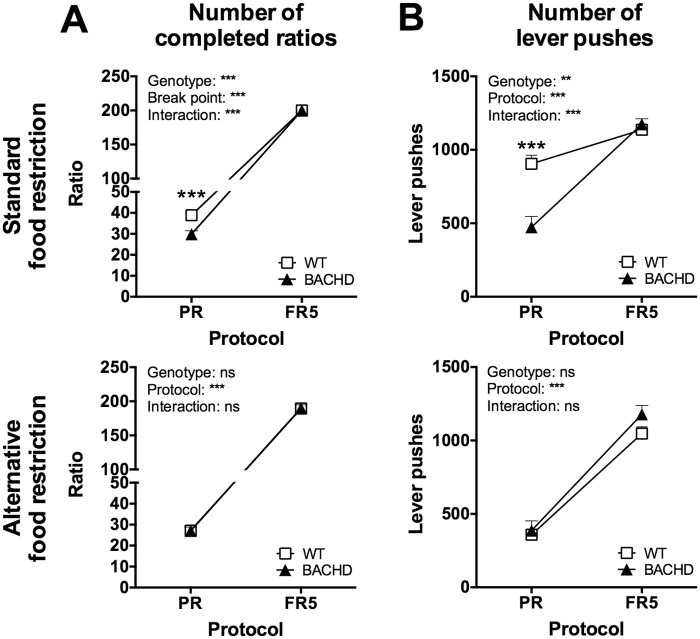
FR5 control test performance during standard and alternative food restriction. The graphs show comparisons of Group I’s performance on the progressive ratio and FR5 control tests, when rats were maintained on the standard and alternative food restriction protocols. The number of completed ratios (A) and number of lever pushes performed on the reinforced lever (B) were analyzed, as detailed break point analysis could not be performed. The graphs display group mean plus standard error. Repeated two-way ANOVA results are displayed inside each graph, and results from *post-hoc* analysis are shown for individual data points in case significant genotype differences were detected. (*P* < 0.05) *, (*P* < 0.01) ** and (*P* < 0.001) ***.

During the last test age, the FR5 control test was repeated when the rats were maintained on the standard and alternative food restriction protocols. During this, most of the rats reached the maximum of 200 reward pellets without making larger breaks, and thus no detailed analysis of break points could be made. Instead, the primary readouts were the number of completed ratios and the number of lever pushes performed on the reinforced lever. Similar to the FR5 test at satiety, there were no differences between BACHD and WT rats in these parameters, and both completed more ratios (Fig 7A) and performed more lever pushes (Fig 7B) compared to their progressive ratio performance.

### Leptin measurements

**Fig 8:**
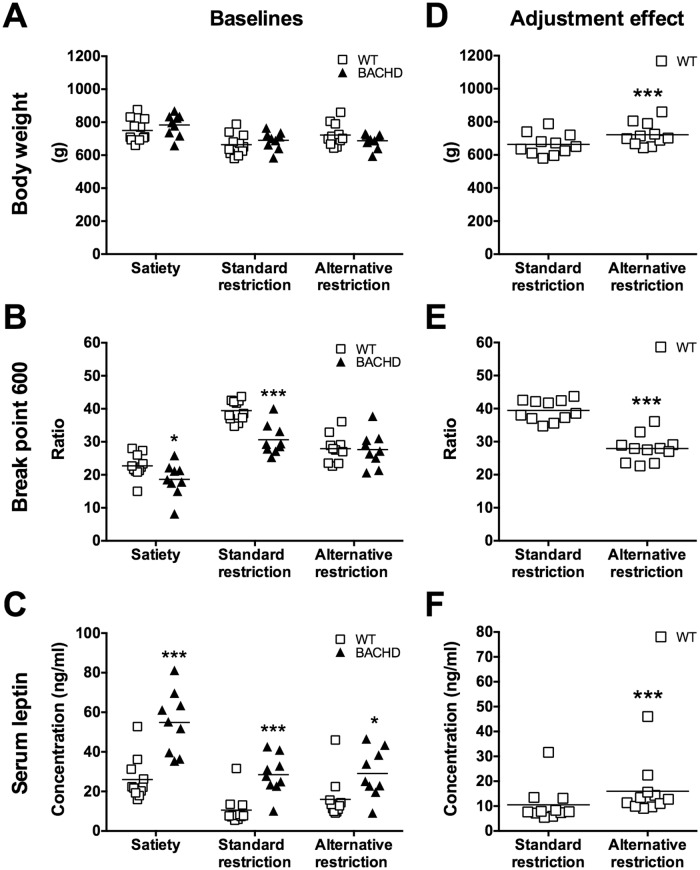
The effect of food restriction adjustment on body weight, progressive ratio performance and serum leptin levels. The graphs show body weight, the number of completed ratios at break point 600 and serum leptin levels of Group I during different food restriction protocols, at 17–19 months of age. (A)–(C) show comparisons between WT and BACHD rats, while (D)–(F) show the specific comparison of WT rats before and after food restriction adjustment. The graphs indicate values from individual rats and group mean. Significant results from *t*-test, Mann-Whitney test, Wilcoxon test or paired *t*-test are displayed inside each graph. (*P* < 0.05) *, (*P* < 0.01) ** and (*P* < 0.001) ***.

BACHD rats showed no significant difference in body weight compared to WT rats at either of the different baselines (Fig 8A), but along with the poorer progressive ratio performance (Fig 8B), they showed significantly higher serum concentrations of leptin (Fig 8C). The difference in leptin levels was strongest at satiety and during the standard food restriction protocol. Although the difference was milder during the alternative food restriction protocol, it was still present. Paired analyses of WT rats further showed that the switch from standard to alternative food restriction resulted in them becoming heavier (Fig 8D) and being less motivated to perform the progressive ratio test (Fig 8E), while having increased serum leptin concentrations (Fig 8F).

### Body composition analysis

**Fig 9:**
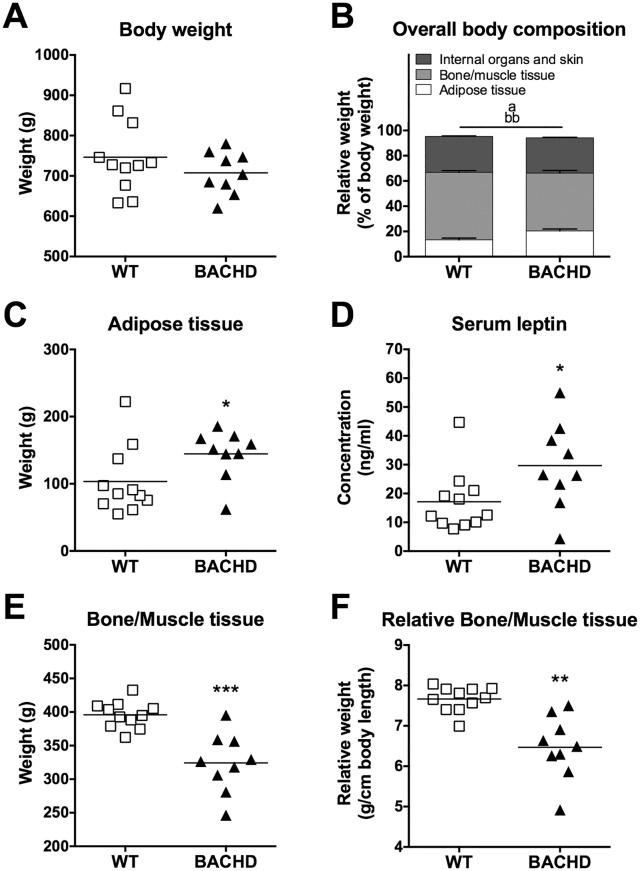
Body composition analysis of rats maintained on alternative food restriction. Parameters of body composition obtained from the dissection of Group I at 19 months of age. Rats were at that time maintained on the alternative food restriction. All graphs except (B) indicate values from individual rats and group mean. (B) indicates group mean plus standard error. Bone/muscle weight in (E) was related to the animals' body lengths to obtain the relative bone/muscle values presented in (F). Significant results from *t*-test or Mann-Whitney tests are displayed inside each graph. (*P* < 0.05) *, (*P* < 0.01) ** and (*P* < 0.001) ***. For (B), "a" denotes a significant difference in adipose tissue (*P* < 0.05) and "bb" denotes a significant difference in bone/muscle tissue (*P* < 0.01).

The detailed dissection of Group I at the study’s endpoint indicated that BACHD and WT rats did not differ in body weight (Fig 9A), but in body composition (Fig 9B). Specifically, BACHD rats carried a larger amount of adipose tissue than WT rats (Fig 9C), displayed higher serum concentrations of leptin (Fig 9D) and had lower absolute and relative bone/muscle tissue mass (Fig 9E and 9F, respectively). Although BACHD rats have regularly been found to be shorter than WT rats in our institute, no significant difference in the total body length was found in this cohort. A trend was, however, present due to the BACHD rats having significantly shorter tails (data not shown).

### Standard food consumption test

**Fig 10:**
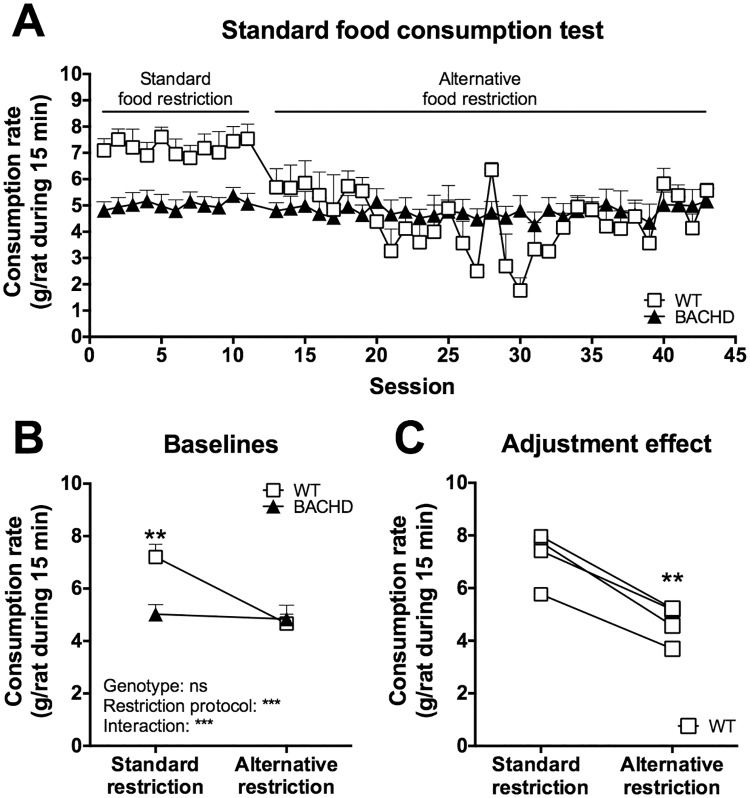
Food consumption rates in the standard food consumption test. Group II's performance in the standard food consumption test at 12 months of age on standard and alternative food restriction is displayed. (A) shows the performance on individual sessions, while (B) and (C) show comparisons of baseline performance during the different food restriction protocols. In (A) and (B) the symbols indicate group mean plus standard error, in (C) the symbols indicate individual WT cages. For (B), repeated two-way ANOVA results are indicated inside the graph, and results from *post-hoc* analysis are displayed in case WT and BACHD differed significantly. For (C), significant results from paired *t*-test is indicated. (*P* < 0.05) *, (*P* < 0.01) ** and (*P* < 0.001) ***.

When the standard food restriction protocol was used, BACHD rats of Group II consistently consumed less food than WT rats in the standard food consumption test (Fig 10A). When WT rats were given more food on a daily basis they responded with reduced food consumption rates (Fig 10A). Through careful adjustments of their feeding regimen it was possible to obtain a setting where they showed comparable food consumption rates to the BACHD rats (i.e. the alternative food restriction protocol) (Fig 10A). Baseline values of the rats’ performance were created, using all sessions performed on the standard food restriction protocol and the last ten sessions performed on the alternative food restriction protocol. Statistical analysis of these baselines showed a clear change in food consumption rate among WT rats due to the adjustment (Fig 10B and 10C). Similar results were obtained for Group I and for several other animal groups that we have assessed (data not shown). Notably, there was no apparent change in the phenotype when the food was placed on the cage floor instead of in the food crib, although rats of both genotypes consumed generally more food in the former setting (S4 Fig).

**Fig 11:**
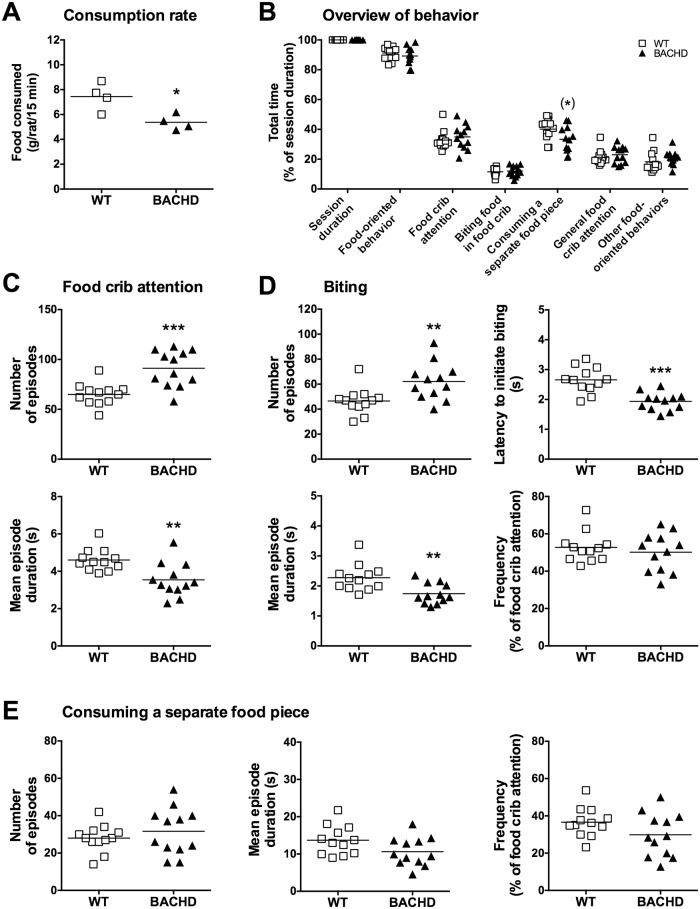
Video scoring of behavioral parameters from the standard food consumption test during standard food restriction. Group II’s performance on the standard food consumption test during the standard food restriction protocol was subjected to detailed video analysis. (A) shows the consumption rate measured for individual cages on the video scored session. (B)–(E) display the behavior of individual rats during the same session. (B) shows the total amount of time spent on different behaviors, in relation to the duration of the test session. (C)–(E) show details concerning some of the behaviors, indicating the number of behavioral episodes, mean episode duration, frequency of behavior and the latency to initiate the behaviors. Frequency relates to the percentage of food crib attention episodes that turn into biting episodes (D) and episodes were rats consume a separate food piece (E). Significant results from *t*-test or Mann-Whitney tests are displayed inside each graph. (*P* < 0.05) *, (*P* < 0.01) ** and (*P* < 0.001) ***. Results in (B) were corrected for multiple comparisons using the Sidak method. Significance levels that were lost through this approach are indicated with a parenthesis.

Detailed video scoring of the rats’ behavior during the standard food consumption test did not indicate any striking differences between WT and BACHD rats when they were maintained on standard food restriction (Fig 11). WT rats consumed more food during the consumption test compared to BACHD rats (Fig 11A), in line with their behavior during baseline performance (Fig 10A). Rats of both genotypes spent comparable amounts of time on arguably food-oriented behaviors, such as paying attention to and biting the food that had been placed in the food crib (Fig 11B). However, further analysis revealed that BACHD rats had a higher number of both food crib attention (Fig 11C) and biting episodes (Fig 11D). These were, however, shorter compared to WT rats’, resulting in the comparable total time spent on either behavior (Fig 11B). Furthermore, BACHD rats had a shorter latency to initiate biting, but there was no difference in how often a food crib attention episode developed into a biting episode (Fig 11D). There was also no difference between genotypes regarding the number of times the rats bit off larger food pieces (Fig 11E). There were, however, trends indicating that BACHD rats took less time to consume such a piece compared to WT rats and that they bit off a separate piece at a slightly lower frequency (Fig 11E). In line with this, there was a significant difference in the total time spent consuming separate food pieces, with BACHD rats spending less time on this activity compared to WT rats (although the difference was no longer significant when multiple comparison corrections were considered) (Fig 11B).

**Fig 12:**
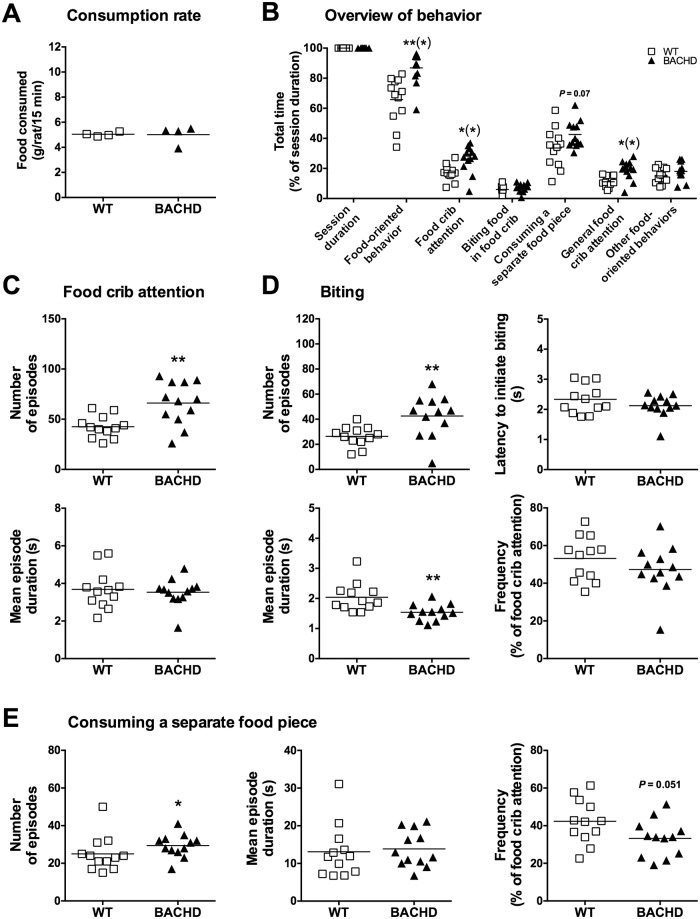
Video scoring of behavioral parameters from the standard food consumption test during alternative food restriction. Group II’s performance on the standard food consumption test during the alternative food restriction protocol was subjected to detailed video analysis. (A) shows the consumption rate measured for individual cages on the video scored session. (B)–(E) display the behavior of individual rats during the same session. (B) shows the total amount of time spent on different behaviors, in relation to the duration of the test session. (C)–(E) show details concerning some of the behaviors, indicating the number of behavioral episodes, mean episode duration, frequency of behavior and the latency to initiate the behaviors. Frequency relates to the percentage of food crib attention episodes that turn into biting episodes (D) and episodes were rats consume a separate food piece (E). Significant results from *t*-test or Mann-Whitney tests are displayed inside each graph. (*P* < 0.05) *, (*P* < 0.01) ** and (*P* < 0.001) ***. Results in (B) were corrected for multiple comparisons using the Sidak method. Significance levels that were lost through this approach are indicated with a parenthesis.

As noted, WT and BACHD rats consumed comparable amounts of food when they were maintained on the alternative food restriction protocol (Fig 12A). Interestingly, under these conditions WT rats spent less time than BACHD rats on food-oriented behaviors (Fig 12B). This was primarily due to them spending less time than BACHD rats on general food crib attention, while the time spent actively biting the food, consuming separate food pieces and performing other food-oriented behaviors did not significantly differ between the genotypes (Fig 12B). BACHD rats still showed a higher number of food crib attention episodes compared to WT rats, although there was no longer any difference in the mean duration of individual episodes (Fig 12C). The rats’ behavior during biting episodes was similar to what was found during the standard food restriction protocol, with BACHD rats showing a higher number of episodes, a shorter mean duration of individual episodes, but no difference in biting episode frequency compared to WT rats. However, in contrast to the previous results, there was no difference between WT and BACHD rats in the latency to initiate biting (Fig 12D). As noted above, BACHD rats spent in total less time than WT rats on consuming separate food pieces when the standard food restriction protocol was used (Fig 11B). An opposite trend was found during the alternative food restriction (Fig 12B and 12E). Specifically, WT rats showed fewer episodes where they consumed separate food pieces compared to BACHD rats (Fig 12E). Interestingly, there was a trend indicating that WT rats still bit off food pieces at a higher frequency (Fig 12E). As before, there was no difference between WT and BACHD rats concerning the mean duration of episodes spent consuming separate food pieces.

In addition to the analysis shown in Figs 11 and 12, a series of curves were made to better display how the WT rats’ behavior changed as a result of the change in food restriction protocol (S5 and S6 Figs). As expected from the results described above, WT rats showed a specific drop in the time spent on food-oriented behavior (S5B Fig) due to a drop in the time spent on general food crib attention (S5E Fig). This in turn appeared to be due to a drop in the mean duration of individual food crib attention episodes, rather than a drop in the number of such episodes (S6A Fig). In line with this, the latency to initiate biting among WT rats was reduced when the alternative food restriction protocol was used (S6B Fig).

### Individual feeding test

**Fig 13:**
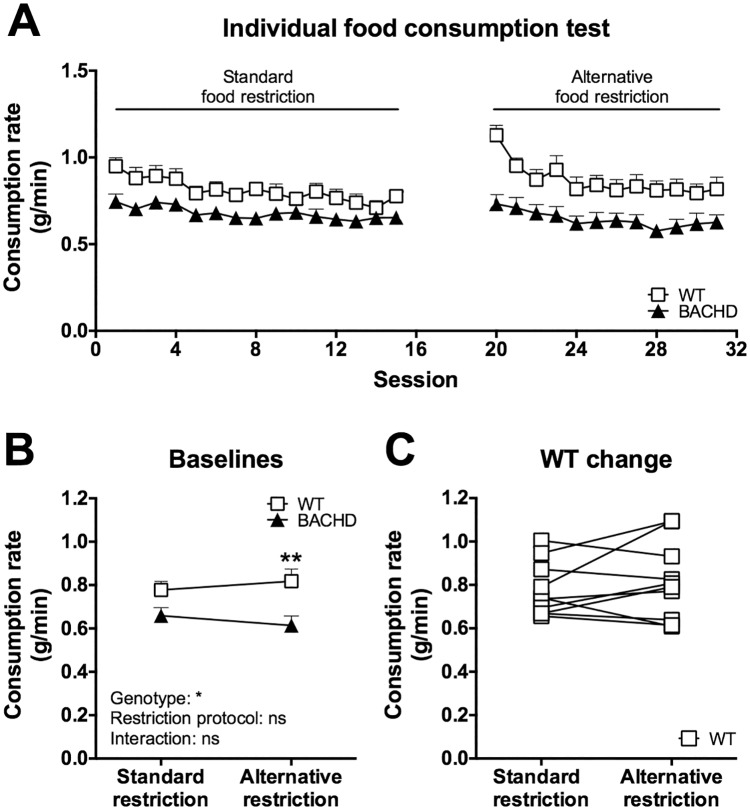
Food consumption rates in the individual food consumption test. Group II's performance in the individual food consumption test at 12 months of age on standard and alternative food restriction is displayed. (A) shows the performance on individual sessions, while (B) and (C) show comparisons of baseline performance during the different food restriction protocols. In (A) and (B) the symbols indicate group mean plus standard error, in (C) the symbols indicate individual WT rats. For (B), repeated two-way ANOVA results are indicated inside the graph, and results from *post-hoc* analysis are displayed in case WT and BACHD differed significantly. For (C), significant results from paired *t*-test is indicated. (*P* < 0.05) *, (*P* < 0.01) ** and (*P* < 0.001) ***.

Most rats reliably consumed the full food piece without frequent or extensive breaks, regardless of which food restriction protocol was used. Two WT rats, however, did not reliably consume the food pellet during the alternative restriction and had to be excluded from the analysis. During both restriction protocols, WT and BACHD rats showed a relatively high consumption rate on initial sessions compared to their stable baseline performance (Fig 13A). For analyzing mean baseline consumption rates, sessions 5–15 and 5–12 were used for the standard and alternative food restriction protocols, respectively. BACHD rats showed a generally lower food consumption rate compared to WT rats during both restriction protocols, although the phenotype was somewhat stronger when the rats were maintained on the alternative food restriction (Fig 13B). The change in food restriction protocol did not appear to have a major impact on the WT rats’ performance (Fig 13C), with the exception of the aforementioned two rats that generally lost interest in consuming the food pellet.

**Fig 14:**
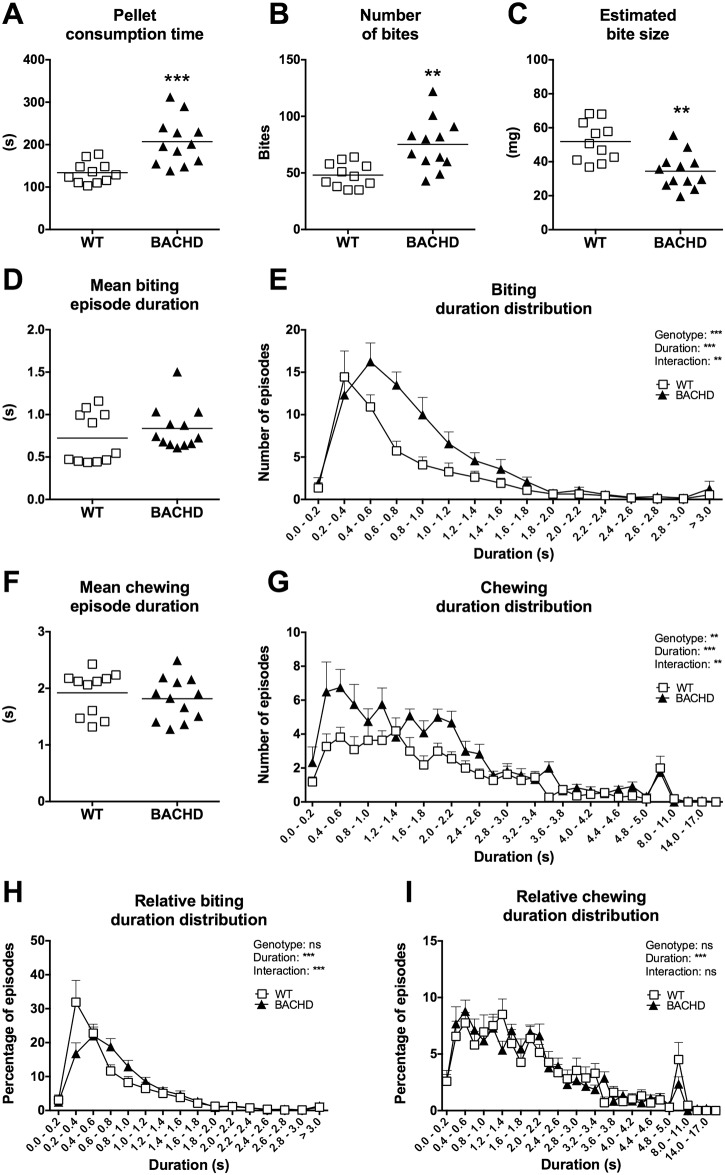
Video scoring of the individual food consumption test during alternative food restriction. Group II’s performance on the first session of the individual food consumption test during the alternative food restriction protocol was subjected to detailed video analysis. (A)–(D) and (F) indicate the performance of individual rats. Significant results from *t*-test or Mann-Whitney test are shown. (E), (G), (H) and (I) show frequency distribution curves for biting and chewing episodes of different durations, indicating group mean plus standard error. The bins used are described in detail in the Material and Methods section. Note that the x-axis in (G) and (I) only labels every other bin. Results from repeated two-way ANOVA are displayed inside the graphs. (*P* < 0.05) *, (*P* < 0.01) ** and (*P* < 0.001) ***.

Video scoring was performed on videos gathered on the first, fifth, sixth and seventh test session of the alternative food restriction test. The first session was chosen due to the phenotype being particularly strong, while session 5–7 were thought to represent baseline performance. Individual biting and chewing episodes were easily identifiable in the videos and made up >96% of the time scored as active feeding (data not shown). The unaccounted time was most likely lost due to the manual nature of the scoring method, which resulted in slight breaks between the scored behaviors whenever a switch between biting and chewing episodes occurred. Video analysis of the first session indicated that BACHD rats needed more time than WT rats to consume the food pellet (Fig 14A). In addition, BACHD rats required more bites compared to WT rats (Fig 14B) and consequently had a smaller estimated bite size (Fig 14C). Although there was no difference in the mean duration of individual biting episodes (Fig 14D), curves showing the biting episode duration distribution still clearly indicated a behavioral difference between the rats (Fig 14E and 14H). While WT rats had a small range of relatively fast bites, BACHD rats showed a slightly right-shifted and broadened peak, indicating that they had slightly longer biting episodes compared to WT rats (Fig 14E and 14H). There was no difference between the genotypes in the mean chewing episode duration (Fig 14F). Detailed analysis of the chewing episode duration distribution indicated that BACHD rats had a higher number of short chewing episodes compared to WT rats (Fig 14G), although the relative distribution of chewing episodes did not indicate any behavioral differences between the genotypes (Fig 14I).

As noted above, the food consumption rate phenotype was noticeably weaker during baseline performance. This was also true for the phenotypes found in the video scoring. BACHD rats still needed more time than WT rats to consume the food pellet (S7A Fig), but there was no longer any statistical difference in the number of bites (S7B Fig) or the estimated bite size (S7C Fig). BACHD rats still showed a shift towards making longer biting episodes compared to WT rats (S7E Fig), although it was less pronounced than during the first test session (Fig 14E). BACHD rats did again not show any indications of having a changed chewing behavior during baseline performance (S7F, S7G and S7I Fig). When splitting the total time needed to consume the food pellet (S8A Fig) into the total time spent biting (S8B Fig) and the total time spent chewing (S8C Fig), BACHD rats spent specifically more time chewing compared to WT rats. Additional analysis of chewing episode distribution, using a different set of bins, indicated that BACHD rats had more chewing episodes of intermediate duration (1.6–5.0 s) compared to WT rats (S8D Fig). BACHD rats also showed an increased total amount of time chewing specifically within this range of chewing episodes (S8E Fig), without showing a difference in mean chewing episode duration (S8E Fig).

Finally, the BACHD rats of Group II were found to have shorter heads compared to their WT littermates (S9A Fig). However, this did not appear to have any major influence on the rats’ food consumption rates (S9C and S9D Fig).

## Discussion

### Progressive ratio performance and motivational phenotype of BACHD rats

One of the aims of the current study was to evaluate if our initial findings concerning the BACHD rats’ performance in the progressive ratio test [18] were reproducible at older ages. This was clearly the case. At all investigated ages, BACHD rats were less motivated than WT rats to perform the test when the standard food restriction protocol was used. When the alternative food restriction protocol was used, WT and BACHD rats reliably showed comparable motivation to perform the test. Ultimately, the results are likely to also be reproducible with other groups of BACHD rats, as they do not appear to be caused by unspecific variations in performance.

Our initial interpretation regarding the motivational deficit in the BACHD rat was that it was likely to be caused by metabolic, rather than psychiatric disturbances [18]. We hypothesized that when the rats were maintained on standard food restriction, WT rats were hungrier than BACHD rats, resulting in them being more motivated to perform lever pushes for a food reward. The alternative food restriction protocol sought to adjust the food restriction levels of the rats, so that they became equally hungry. As this reliably resolved the motivational deficit in the progressive ratio test, we considered it unlikely that the initial phenotype had been caused by psychiatric deficits. In the current study, we aimed at further evaluating this idea by performing the progressive ratio test while the rats had free access to food. This constituted a second feeding condition (on top of the alternative food restriction), where WT and BACHD rats should be equally hungry (i.e. in this case satiated). However, in contrast to their behavior during the alternative food restriction, BACHD rats were found to be less motivated than the WT rats in this setting (i.e. similar to the rats’ behavior during the standard food restriction). Importantly, this did not appear to be due to BACHD rats becoming satiated or fatigued at an earlier point than WT rats, as performance on the FR5 control test (where rats of both genotypes performed more pushes and consumed more pellets compared to the progressive ratio sessions) did not differ between the genotypes (for the same reason, the BACHD rats’ reduced motivation during the standard food restriction is likely not caused by fatigue or satiety). Ultimately, a difference in hunger levels is unlikely to fully explain the motivational deficit found in BACHD rats performing the progressive ratio test. Still, the phenotype might be otherwise connected to the rats’ metabolic disturbances.

As previously noted, male BACHD rats are obese [18]. Leptin, an endocrine hormone secreted from white adipose tissue [23], has been shown to affect rats’ motivation to perform the progressive ratio test. Specifically, increased leptin signaling has been found to reduce motivation [24,25], while knock-down of leptin receptors has been found to increase motivation [26]. Interestingly, leptin has been shown to decrease motivation in progressive ratio tests both at satiety [24,25] and during food restriction [26]. Because of this, we hypothesized that the motivational deficit seen in BACHD rats during satiety and the standard food restriction was caused by an obesity-related increase in serum leptin levels. We further hypothesized that this phenotype would be resolved through the use of the alternative food restriction protocol. To evaluate this, we measured serum leptin levels in Group I during their different performance baselines. The results clearly indicated that BACHD rats had higher leptin levels than WT rats both at satiety and when the standard food restriction protocol was used. However, although the difference was less apparent during the alternative food restriction, it was not fully resolved. In line with this, the dissection results clearly showed that the BACHD rats still carried more adipose tissue than WT rats when they were maintained on the alternative food restriction. Thus, the results appear to argue against the hypothesis that the BACHD rats’ motivational deficit is caused primarily by their obesity. Still, it is not known how large the difference in leptin levels would have to be in order to result in such a phenotype. In relation to this, it is also unknown, if the neuronal circuits necessary for leptin signaling are intact in BACHD rats. To better understand the current results, it would therefore be of interest to investigate dose-response curves for leptin’s effect on BACHD and WT rats’ progressive ratio performance. In addition, it would be important to study the expression of leptin receptors in the BACHD rats’ mid- and hindbrain, as these regions appear to be involved in progressive ratio performance [24,26,34] (interestingly, leptin receptors in the hypothalamus appear to be of less importance [26]).

However, further studies of the integrity of the BACHD rats’ leptin system are unlikely to offer any final conclusions regarding whether or not their motivational deficit is caused by their obesity. For this, efforts should be made to elucidate the cause of the rats’ obesity, so that lean BACHD rats might be obtained and subsequently assessed in the progressive ratio test. Interestingly, inactivating mutant huntingtin expression in the hypothalamus of BACHD mice completely resolved their obesity phenotype [35]. Although the cause for the obesity phenotypes might differ between BACHD mice and BACHD rats (obesity in the mouse model has been suggested to be due to overeating [35], while this does not appear to be the case in the rat model [17,18]), both could be due to hypothalamic pathologies, involving different subregions [36]. Regarding BACHD rats, the arcuate nucleus is particularly interesting, as lesioning this region has been shown to result in obesity without associated hyperphagia [36–40]. Interestingly, the lesions appear to target neuron populations that are involved in regulating the release of growth hormone [40–43]. In line with this, down-regulation of growth hormone signaling has been found to result in growth impairments coupled with obesity [44–46], i.e. specifically the phenotypes that we have previously noted in male BACHD rats [18]. Moreover, one of the peripheral functions of growth hormone is to stimulate the release of IGF-1 from the liver [47], and we have repeatedly found that male BACHD rats have lower serum levels of IGF-1 (unpublished data). Thus, the growth hormone signaling axis and the integrity of the arcuate nucleus are of great interest for future work with the BACHD rats. In connection to this, detailed investigation of the similarities and differences in male and female BACHD rats’ physiologies would be of importance.

The phenotype of reduced motivation among BACHD rats remained arguably stable when the rats were retested at older ages. Still, there were some indications that a subtle progressive worsening of the phenotype might be present (i.e. the *post-hoc* analysis shown in Fig 2B and the results from the three-way ANOVA Genotype x Age interaction effect). However, additional longitudinal studies of the BACHD rats’ progressive ratio performance would be necessary to conclude if this is truly the case. Based on HD’s clinical presentation, one would expect disease-related phenotypes in animal models to progressively worsen with age. In line with this, other phenotypes found in the BACHD rats have shown strong progressive change even at ages below four months [17,48,49], which is well within the ages investigated in the current study. Still, it is worth noting that the obesity phenotype did not appear to change with age during our previous study [18], so if that indeed causes the motivational phenotypes one would expect the latter to remain reasonably stable as well. Still, not all psychiatric symptoms in HD patients clearly progress with age either [50]. For example, while apathy appears to progressively worsen, depression does not [22,50–52]. Performance in the progressive ratio test at satiety has been suggested to be primarily affected by the rats’ hedonic value of the food reward, while motivation to perform the test during food restriction is thought to be more governed by the induced energy imbalance (i.e. hunger) [53, 54]. As the BACHD rats were less motivated to perform the test at satiety, it is possible that their motivational phenotype is at least to some extent due to anhedonia, which is an aspect of depression that has been implicated in HD [55]. In the end, the apparent lack of progression seen in the BACHD rats’ motivational deficit does not offer any clear insight into the specific nature of the phenotype.

Although the alternative food restriction protocol only changed the WT rats’ restriction conditions, BACHD rats also showed drops in motivation between the performance baselines established on the standard and alternative restriction protocols. During the 7–9 months test, this was primarily caused by the set of prefeeding tests described in the Material and methods section. As noted, both WT and BACHD rats failed to return to their initial performance baseline between the prefeeding tests, and their motivation instead dropped with each session. As the prefeeding tests were run between the establishment of performance baselines on the standard and alternative restriction, the BACHD rats show a clear drop in motivation when the two are compared directly. As the same issue concerned the WT rats, the change in restriction protocol appeared to have a particularly pronounced effect on performance during the 7–9 months test (as indicated by the significant Restriction protocol x Age interaction effect revealed by the three-way ANOVA). The drop in motivation that was seen among BACHD rats during the later test ages was instead likely related to a specific aspect of the food restriction. As noted, both the standard and alternative food restriction protocols took natural growth into account, which meant that the amount of food given to the rats was continuously adjusted. We have in other studies found that the current calculations result in a slight over-correction of the food restriction (due to the expected growth being overestimated). The error increases with experiment duration, although we have not found any strong behavioral effects of this and have reliably been able to establish stable performance baselines. Still, this is likely the reason for the small drop in motivation seen among BACHD rats when directly comparing their baselines from the current study.

One final and important aspect to consider in the current progressive ratio results concerns the readouts that were not directly related to the rats’ motivation, as these indicated that BACHD rats might suffer from striatal impairments. First, there is the slowed food pellet retrieval seen among BACHD rats. From the several Skinner box-based tests that we have run so far at our institute, this phenotype is found in almost all test protocols and animal groups (largely unpublished, but see [18]). Thus, it offers an interesting and reproducible phenotype to work with, although it is at this point unclear if the impairment is caused by motoric or psychiatric deficits. Similar phenotypes have been found in the TgHD rat model of HD [56] and rats with lesions to the dorsolateral striatum [57]. Second, BACHD rats were found to perform an increased number of excessive (i.e. perseverative) lever pushes. This has also been seen in rats with lesions to the dorsal striatum [57]. Interestingly, such lesions do not appear to affect the rats’ overall motivation to perform the progressive ratio test [57]. Thus, the slowed pellet retrieval latency and the increase in perseverative lever pushes suggest that the BACHD rats suffer from some kind of striatal dysfunction, which is likely separate from what causes their motivational impairment. In line with this, the slowed pellet retrieval and perseverative responding were present on both standard and alternative food restriction.

### Food consumption rate phenotypes of BACHD rats

In our initial study [18], we used a food consumption test (the standard food consumption test) in order to estimate the rats’ apparent hunger and food interest, as similar methods had been used by others [28–33]. In the current study, we sought to extend our initial work by adding a video-based scoring of the rats’ behavior in the standard food consumption test, and also assess how they consume individual food pieces (individual food consumption test). When the standard food restriction protocol was used, BACHD rats were found to have a lower food consumption rate compared to WT rats in both tests. In contrast, when the alternative food restriction protocol was used, there was no difference in the rats’ food consumption rate in the standard food consumption test, while BACHD rats were still slower than WT rats in the individual food consumption test.

BACHD rats were found to require more biting episodes than WT rats in order to consume the food pellets in the individual food consumption test. As all rats were given food pellets of comparable size, the results suggest that BACHD rats also took smaller bites compared to WT rats. This phenotype could be related to BACHD rats having problems with biting larger pieces off from the food pellet, with keeping a large amount of food inside their mouths or with efficiently chewing and swallowing a large food piece. Importantly, the deficit did not appear to be due to the BACHD rats’ smaller heads, and did not seem to be strongly influenced by hunger. In addition to requiring a higher number of biting episodes to consume the pellets, the BACHD rats’ biting episodes were slightly longer than the WT rats’. It is possible that BACHD and WT rats used similar techniques for biting pieces off from the food pellet. If so, the BACHD rats’ longer biting episodes might indeed indicate that they had problems biting pieces off. However, the results might also be due to BACHD rats preferring more time-consuming techniques (such as gnawing rather than performing distinct bites) compared to WT rats. More detailed scoring would be required to determine if that was the case. Further characterization work would also be necessary in order to determine if this could explain the BACHD rats’ smaller bite size, and whether or not it would be related to a motoric impairment. The duration of single biting episodes among both WT and BACHD rats were still short compared to the chewing episodes. Therefore, the latter likely had a stronger impact and probably contributed more to the BACHD rats’ food consumption rate phenotype. In line with the hypothesis that BACHD rats made smaller bites compared to WT rats, analysis revealed that they showed a higher absolute (but not relative) number of short chewing episodes. If BACHD rats were as skillful as WT rats at chewing and swallowing, while managing smaller volumes of food during each chewing episode, one might have expected them to show a more pronounced shift towards shorter chewing episodes. The apparently unchanged frequency distribution of chewing episodes could thus indicate that BACHD rats indeed have problems with chewing and swallowing. In this regard, it is worth considering that the smaller bite size discussed above might constitute a compensatory mechanism, allowing BACHD rats to maintain optimal (i.e. seemingly unchanged) chewing. Impaired chewing and swallowing could be due to motoric impairments, although other possibilities should also be considered. Specifically, we have found that BACHD rats have disproportionally small salivary glands (unpublished results), which might impair their ability to form a convenient food bolus. HD patients often suffer from problems regarding eating, with particularly frequent problems when swallowing [58–61]. We have repeatedly performed tests where WT and BACHD rats are allowed to consume a large amount of the reward pellets used in the Skinner boxes (see [18] for a published example). Typically, BACHD rats are slightly slower than WT rats during initial sessions of this, but quickly reach a comparable consumption rate. Importantly, consumption of these small reward pellets appears to involve very limited chewing behavior, suggesting that other aspects (such as tongue protrusion and swallowing) are more important determinants of the food consumption rate in this test. Given the BACHD rats’ generally unimpaired performance in these tests, their ability to swallow is most likely not strongly impaired. In addition, we have performed several tests where BACHD rats were allowed to consume spaghetti pieces (unpublished data). Feeding behavior under these circumstances appears to involve biting primarily with the incisors, and once again limited chewing. Again, BACHD rats have generally been found to show comparable consumption rates to WT rats in this test. Thus, the key factor causing the BACHD rats’ slowed food consumption in the individual food consumption test might concern the test’s strong dependency on chewing and/or the formation of a convenient bolus for swallowing. Further efforts should be made to characterize the noted consumption rate deficit, as it constitutes an interesting and robust phenotype seen among the BACHD rats.

Our initial interpretation of the slowed consumption rate among BACHD rats in the standard food consumption test was that they were less hungry compared to WT rats, and that the alternative restriction protocol resolved this difference. The results from the current study do not strongly support this idea, but do not necessarily refute them either. When the standard restriction protocol was used, BACHD and WT rats generally behaved in a comparable way. They showed similar amounts of food-oriented behaviors and spent the same amount of time on both paying attention to the food in the food crib and actively trying to bite pieces off from it. As it is clear that BACHD rats still consumed less food than WT rats, it is reasonable to assume that their biting behavior was less efficient. The analysis also indicated that BACHD rats performed more but shorter biting episodes compared to WT rats. Based on the results from the individual food consumption test, it seems fair to assume that this might be due to them taking a high number of smaller bites, while WT rats made a low number of larger bites. This is further supported by the fact that episodes where rats consumed a separate food piece did not differ in length between the genotypes. If BACHD and WT rats had bitten off pieces of comparable size, one would expect these consumption episodes to be longer among BACHD rats (according to the results of the individual food consumption test). Thus, the nature of the reduced food consumption rate among BACHD rats seems at first glance to be comparable between the standard and individual food consumption tests. Ultimately, this suggests that the phenotype seen in the standard food consumption tests during standard food restriction is primarily due to them taking smaller bites, which (based on the results from the individual food consumption test) might not be strongly affected by hunger.

The alternative food restriction protocol sought to match WT and BACHD rats’ food consumption rates (with the assumption that this represented the rats’ hunger level). This primarily focused on giving more food to WT rats, and as a consequence, there was a clear change in their behavior. Most notably, the amount of time spent on food-oriented behaviors and paying attention to the food crib dropped below the level of BACHD rats. This appeared to be largely a result of WT rats making shorter visits to the food crib, rather than fewer. This, in turn, seemed to be due to the WT rats showing a reduced latency to initiate biting episodes. In contrast, the time spent biting at the food remained unchanged and comparable to both their behavior during the standard food restriction protocol and to that of the BACHD rats. As the WT rats still consumed less food under these circumstances, the results suggest that their biting behavior had now become less efficient. Due to the limitations of the video quality, it remains unclear if the unidentified hunger-sensitive behaviors that modulated the WT rats’ biting efficiency were the same as the ones causing the BACHD rats’ reduced food consumption rate during the standard food restriction protocol. As noted, a reduced bite size might be the cause of the BACHD rats’ reduced consumption rate in the standard food consumption test. A change in bite size could theoretically also explain the change in the WT rats’ consumption rate during the alternative food restriction. The latter is, in contrast to the former, not supported by the results from the individual food consumption test, as bite size appeared to be unaffected by the change in food restriction protocol. Still, it should be noted that food consumption behavior in these two tests might not be directly comparable. In the standard food consumption test, the rats remain in their home cage and are allowed to consume food if they are interested. In contrast, in the individual food consumption test the rats are more or less forced to consume the food piece before being allowed to return to their home cage. Thus, the latter test might have conditioned the rats to eat as fast as possible, rather than based on how hungry they were. This might have resulted in the evaluated parameters’ (e.g. bite size) apparent resistance to a change in hunger levels. Importantly, it is clear that hunger was not the only factor that affected the rats’ performance in the individual consumption test, as both WT and BACHD rats showed very high consumption rates during early sessions and needed several sessions to approach a stable baseline performance. This was despite the fact that the rats were maintained on a constant feeding regimen. Thus, while bite size appears to be unaffected by hunger in the individual food consumption test, it might still be sensitive to hunger in the standard food consumption test. Ultimately, it is therefore still possible that the less efficient biting behavior of BACHD rats in the standard food consumption test during standard food restriction is caused by them being less hungry compared to WT rats. Additional work is needed before a final conclusion regarding this matter can be reached.

The more extensive investigation of WT and BACHD rats’ food consumption behavior in the current study was in part performed to better understand the progressive ratio phenotype of the BACHD rats. As noted, the BACHD rats’ motivational deficit cannot be fully explained by a difference in hunger or leptin levels. Based on the results above, it can be further argued that the standard food restriction constitutes a suitable protocol, as it seems to induce similar food interest among WT and BACHD rats. In addition, one can argue that the lower food consumption rates among BACHD rats could primarily be caused by non-hunger related differences in feeding behavior. If so, the alternative food restriction protocol would only serve to mask the underlying phenotype rather than to resolve it. This would also suggest that the apparent lack of a motivational deficit in the progressive ratio test during the alternative food restriction is coincidental. Ultimately, the true phenotype of the BACHD rats would be a reduced motivation to perform the progressive ratio test, likely based on a psychiatric deficit. However, as noted above, the influence of the BACHD rats’ obesity and increased leptin levels on their progressive ratio performance is not clear, and conclusive results still have to be obtained. Likewise, the exact nature of the food consumption rate phenotype in the standard food consumption test remains unclear, and could still involve more discreet hunger-related behaviors than the ones scored here.

### Connection to previously noted motor impairments of the BACHD rats

Other studies have sought to directly investigate the presence of motor impairments among the BACHD rats [17,48,49]. These have revealed early (onset at one to two months of age) progressive deficits in the BACHD rats’ ability to maintain balance on a rotating rod [17,48,49], and late (onset at twelve to fourteen months) deficits in unhindered gait [17,48]. The results from the current study suggest that yet another kind of motor function (i.e. orofacial) might be disturbed in the BACHD rat, and is worth investigating further. From the results we have gathered so far, these impairments appear to show an early onset [18], without any clear progression with age. All together, the results suggest that BACHD rats might suffer from a range of different motor impairments, which become apparent and progress differently throughout their disease development. However, the influence that possible confounding factors (such as repeated exposure to the stressful rotarod test and the BACHD rats’ changed physiology) have had on these motor impairments has not been investigated. Thus, additional work is needed before conclusions on the overall picture of the BACHD rats’ motor impairments can be drawn.

### Assessing BACHD rats’ performance in food-based tests

One of the overarching aims of our research is to investigate the presence of cognitive impairments in the BACHD rat. A large concern when considering this has been the BACHD rats’ metabolic phenotypes and the possibility that these could confound the readouts of a given behavioral protocol. Although it remains unclear if the obesity phenotype is the main cause of the BACHD rats’ reduced motivation to perform the progressive ratio test, the consistent difference in motivation is of importance when considering other behavioral protocols. Notably, differences in motivation have been found to result in remarkable differences in behavior [20]. In our initial publication [18], we argued that the alternative food restriction protocol constitutes a good approach to achieve an experimental setting where WT and BACHD rats are comparably motivated to perform a given food-reinforced behavioral test. Although the current results also largely argue for that, the use of the alternative food restriction protocol can no longer be fully supported. This is primarily due to the fact that it is based on matching the rats’ food consumption rates, with the assumption that this represents a good measurement of hunger. As noted, the exact nature of the BACHD rats’ reduced food consumption rates is not clear, and it might be influenced by non-hunger related feeding impairments. However, the standard food restriction protocol clearly results in WT and BACHD rats having different metabolic characteristics, and would likely result in them being differently interested in performing a given food-reinforced test. As neither protocol is optimal on its own, we suggest that any behavioral characterization performed with BACHD rats in food-reinforced tests should include appropriate control tests. These should aim at investigating how the readouts of the given test are affected by changes in motivation. If phenotypes are found in parameters that are sensitive to changes in motivation, interpretations should be made carefully.

Another option is to use cognitive tests that do not rely on food reinforcements. Specifically, tests that make use of larger maze setups frequently use the possibility of returning to the home cage as an incentive for rats to perform the given task. Such a protocol has previously been used for evaluating reversal learning in BACHD rats [62] (the authors specifically argued that avoiding food restriction would be preferable when considering the difference in *ad libitum* food consumption first described in [17]). Still, this should also be done with some caution, as BACHD rats have repeatedly been found to show reduced anxiety in a test of exploration behavior [17,49]. Such a phenotype might under some circumstances result in them having a reduced interest in returning to their home cage compared to WT rats. Thus, further investigation of the use of this kind of reinforcement should also be made before considering it a better alternative.

### Conclusions and final remarks

The current study does not offer any final conclusions regarding the reduced motivation and food consumption rate found among male BACHD rats. It does, however, support the results of our initial study [18], indicating that BACHD rats are likely to be less motivated than WT rats to perform food-reinforced tasks when standard food restriction protocols are used.

In addition, detailed analysis of progressive ratio performance revealed that BACHD rats were reliably slower at retrieving the reward pellets, and had an increased tendency to perform excessive lever pushes. Both phenotypes appeared to be unrelated to their lower motivation, and might be indicators of striatal dysfunction.

We further found clear indications that male BACHD rats are slower than WT rats in consuming single pieces of standard rodent chow, suggesting a non hunger-related feeding impairment reminiscent of eating problems in HD patients. Because of this, we no longer consider it advisable to use the standard food consumption test as a test of hunger when working with BACHD rats.

As the presence of motivational differences between WT and BACHD rats is a possible confounding factor when working with food-based tests, and as the alternative food restriction protocol is not necessarily better than the standard restriction protocol, we suggest that any work with BACHD rats and food-reinforced tests should include appropriate control tests.

## Supporting information

S1 Fig Pushes on the non-reinforced lever during the progressive ratio test. Age progression of the number of pushes on the non-reinforced lever during the progressive ratio test performed with Group I is shown. (A) shows performance during the standard food restriction protocol, while (B) shows performance during the alternative food restriction protocol. The graphs indicate group mean plus standard error. Repeated two-way ANOVA results are displayed in each graph, and results from *post-hoc* analysis are shown for individual data points in case significant genotype differences were detected. (*P* < 0.05) *, (*P* < 0.01) ** and (*P* < 0.001) ***.
(TIFF)

S2 Fig FR5 phase of the progressive ratio test on standard and alternative food restriction. Group I’s performance during the FR5 phase of the progressive ratio test, during both food restriction protocols, is shown. Data was created based on the overall performance on all test ages, as no consistent change with age was found for the parameters. Detailed information on how the different parameters were measured is given in the Material and Methods section. (A) indicates the performance of individual rats and group mean. Significant results from Mann-Whitney test are shown inside the graphs. (B)–(D) show group mean plus standard error. Repeated two-way ANOVA results are displayed inside the graphs, and results from *post-hoc* analysis are shown for individual data points in case significant genotype differences were detected. (*P* < 0.05) *, (*P* < 0.01) ** and (*P* < 0.001) ***.
(TIFF)

S3 Fig The effect of food restriction on break point 600. The graphs show comparisons of the number of ratios completed at break point 600 for Group I during their progressive ratio baselines at satiety and the standard food restriction protocol. (A) shows data from the tests performed at 12–14 months of age. (B) shows data from the tests performed at 17–19 months of age. The curves indicate group mean plus standard error, repeated two-way ANOVA results are displayed inside the graphs, and results from *post-hoc* analysis are shown for individual data points in case significant genotype differences were found. (*P* < 0.05) *, (*P* < 0.01) ** and (*P* < 0.001) ***.
(TIFF)

S4 Fig Performance in the standard food consumption test using different food placements. When Group II was maintained on the standard food restriction protocol, one session of the standard food consumption test was run with the food placed inside of the cage (on the cage floor) instead of in the food crib. Data from this session is compared to the performance baseline of the standard food consumption test. The curve indicates group mean plus standard error, repeated two-way ANOVA results are displayed inside the graph. *Post-hoc* analysis did not reveal significant genotype differences. (*P* < 0.05) *, (*P* < 0.01) ** and (*P* < 0.001) ***.
(TIFF)

S5 Fig Effect of the change in food restriction protocol on the behavior in the standard food consumption test (part I). The graphs show the change in Group II’s behavior in the standard food consumption test, when the food restriction protocol was changed from the standard to the alternative approach. Graphs indicate group mean plus standard error. (A) displays results from repeated two-way ANOVA inside the graph and *post-hoc* analysis at data points where performance between the genotypes differed significantly. (B)–(G) concern the total amount of time spent on the different scored behaviors, and show significant results from *t*-test or Mann-Whitney test for single comparisons between the genotypes on either restriction protocol (see also Figs 10B and 11B). (*P* < 0.05) *, (*P* < 0.01) ** and (*P* < 0.001) ***.
(TIFF)

S6 Fig Effect of the change in food restriction protocol on the behavior in the standard food consumption test (part II). The graphs show the change in Group II’s behavior in the standard food consumption test, when the food restriction protocol was changed from the standard to the alternative approach. Graphs indicate group mean plus standard error. The graphs concern details regarding the number of behavioral episodes, their mean duration, frequency and initiation latency of the different scored behaviors. Significant results from *t*-test or Mann-Whitney test for single comparisons between the genotypes on either restriction protocol are shown (see also Figs 10C–10E and 11C–11E). (*P* < 0.05) *, (*P* < 0.01) ** and (*P* < 0.001) ***.
(TIFF)

S7 Fig Video scoring of individual food consumption test baseline during alternative food restriction protocol. Group II’s mean performance on session 5–7 of the individual food consumption test during the alternative food restriction protocol was subjected to detailed video analysis in order to investigate baseline behavior. (A)–(D) and (F) indicate the performance of individual rats. Significant results from *t*-test or Mann-Whitney test are shown in case significant genotype differences were found. (E), (G), (H) and (I) show frequency distribution curves for biting and chewing episodes of different durations, indicating group mean plus standard error. The bins used are described in detail in the Material and Methods section. Note that the x-axis in (G) and (I) only labels every other bin. Results from repeated two-way ANOVA are displayed inside the graphs. (*P* < 0.05) *, (*P* < 0.01) ** and (*P* < 0.001) ***.
(TIFF)

S8 Fig Further analysis of the performance difference found in the individual food consumption test. Group II’s performance on session 5–7 of the individual food consumption test during the alternative food restriction protocol was subjected to detailed video analysis in order to investigate baseline behavior. As the initial analysis of these sessions (see S7 Fig) did not clearly reveal the same phenotypes as found in the first session (see Fig 14), additional parameters were analyzed. These particularly concerned the total time spent biting (B) and chewing (C) the food, as well as the frequency distribution of chewing episodes of different durations, using different bins (E) (compare to Fig 14E, 14I and S7E, S7I Fig). Graphs indicate the performance of individual rats and group mean. Significant results from *t*-test or Mann-Whitney test are shown. (*P* < 0.05) *, (*P* < 0.01) ** and (*P* < 0.001) ***.
(TIFF)

S9 Fig Head size phenotype and its influence on the individual food consumption test. The head size of the rats in Group II was measured at the endpoint of the study, and a brief analysis was made to evaluate if this parameter had any strong influence on the rats' performance. For this, the food consumption of a subgroup of rats with comparable head size was investigated. As noted in previous studies [18], BACHD rats were found to have smaller heads than WT rats (A). (B) displays the comparable head sizes in the subgroup used for further analyses. (C) displays the mean food consumption rates of both the full groups and the subgroups with comparable head sizes (see also Fig 12). (D) shows the mean food consumption rate during baseline performance for the subgroup. (A), (B) and (D) indicate data from individual rats. (C) indicates group mean plus standard error. For (A), (B) and (D), significant results from *t*-test or Mann-Whitney test are shown. (*P* < 0.05) *, (*P* < 0.01) ** and (*P* < 0.001) ***.
(TIFF)
