# Correction: Further investigation of phenotypes and confounding factors of progressive ratio performance and feeding behavior in the BACHD rat model of Huntington disease

**DOI:** 10.1371/journal.pone.0213755

**Published:** 2019-03-07

**Authors:** Erik Karl Håkan Clemensson, Laura Emily Clemensson, Benedikt Fabry, Stefanie Flunkert, Olaf Riess, Robert Wronski, Huu Phuc Nguyen

Dr. Stefanie Flunkert should be included in the author byline. She should be listed as the fourth author, and her affiliation is 3: QPS Austria, Grambach, Austria. The contributions of this author are as follows: Conceptualization, Funding Acquisition, Project Administration, and Supervision.

Dr. Robert Wronski should also be included in the author byline. He should be listed as the sixth author, and his affiliation is 3: QPS Austria, Grambach, Austria. The contributions of this author are as follows: Conceptualization, Funding Acquisition, Project Administration, and Supervision.

The correct citation is: Clemensson EKH, Clemensson LE, Fabry B, Flunkert S, Riess O, Wronski R, et al. (2017) Further investigation of phenotypes and confounding factors of progressive ratio performance and feeding behavior in the BACHD rat model of Huntington disease. PLoS ONE 12(3): e0173232. https://doi.org/10.1371/journal.pone.0173232
