# Supplementary figures and images for: Further investigation of phenotypes and confounding factors of progressive ratio performance and feeding behavior in the BACHD rat model of Huntington disease
Source: PLoS One. 2017 Mar 8;12(3):e0173232. doi: 10.1371/journal.pone.0173232 (PMC5342229; doi:10.1371/journal.pone.0173232)

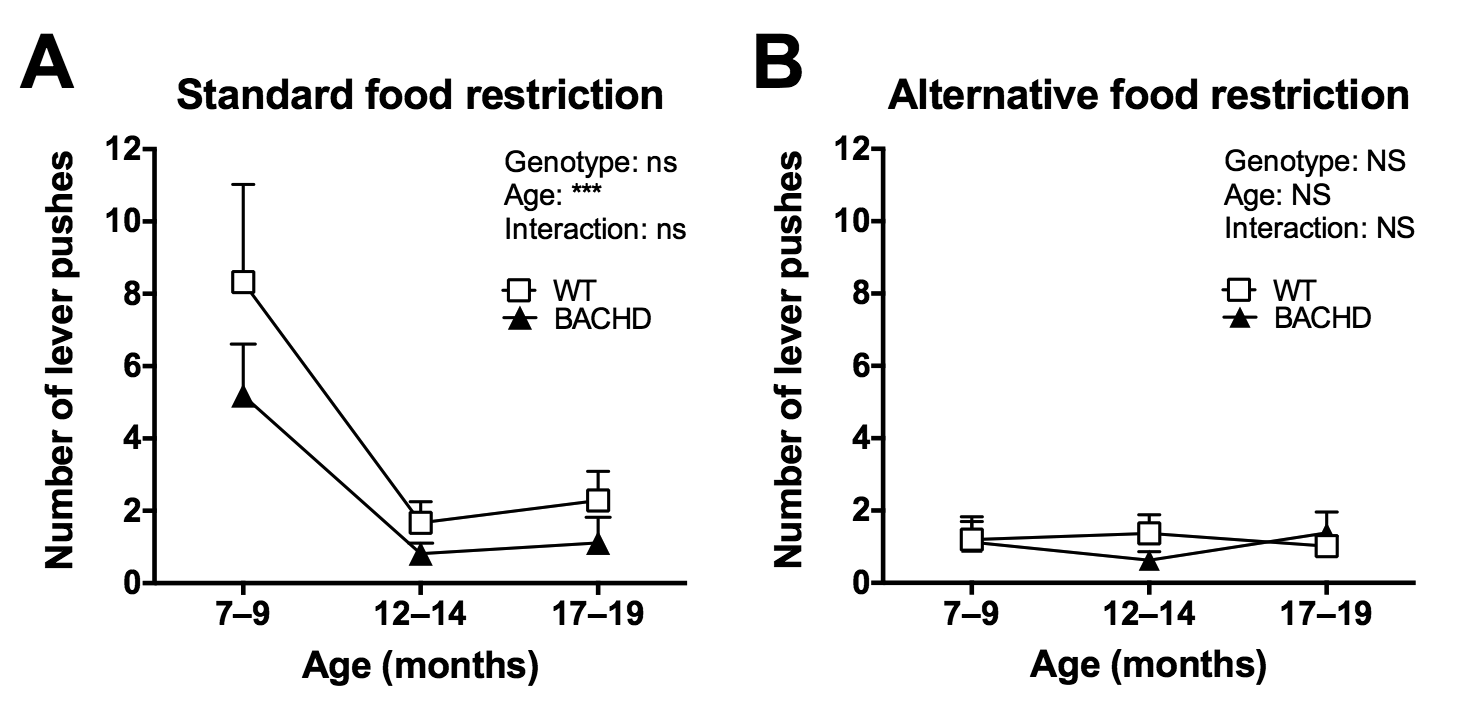

Supplement: S1 Fig — Age progression of the number of pushes on the non-reinforced lever during the progressive ratio test performed with Group I is shown. (A) shows performance during the standard food restriction protocol, while (B) shows performance during the alternative food restriction protocol. The graphs indicate group mean plus standard error. Repeated two-way ANOVA results are displayed in each graph, and results from post-hoc analysis are shown for individual data points in case significant genotype differences were detected. (P < 0.05) *, (P < 0.01) ** and (P < 0.001) ***. (TIFF) [file pone.0173232.s001.tiff]

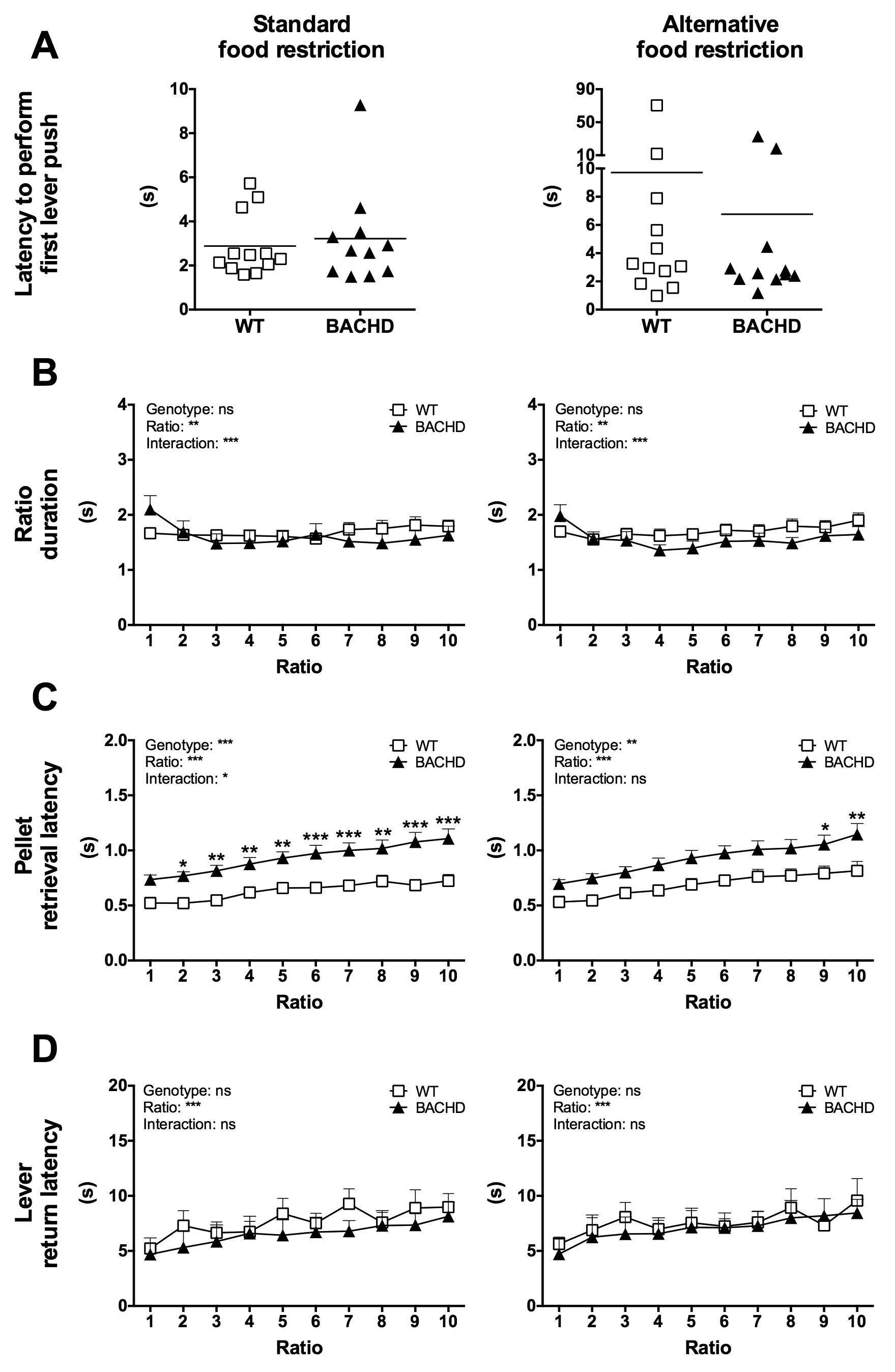

Supplement: S2 Fig — Group I’s performance during the FR5 phase of the progressive ratio test, during both food restriction protocols, is shown. Data was created based on the overall performance on all test ages, as no consistent change with age was found for the parameters. Detailed information on how the different parameters were measured is given in the Material and Methods section. (A) indicates the performance of individual rats and group mean. Significant results from Mann-Whitney test are shown inside the graphs. (B)–(D) show group mean plus standard error. Repeated two-way ANOVA results are displayed inside the graphs, and results from post-hoc analysis are shown for individual data points in case significant genotype differences were detected. (P < 0.05) *, (P < 0.01) ** and (P < 0.001) ***. (TIFF) [file pone.0173232.s002.tiff]

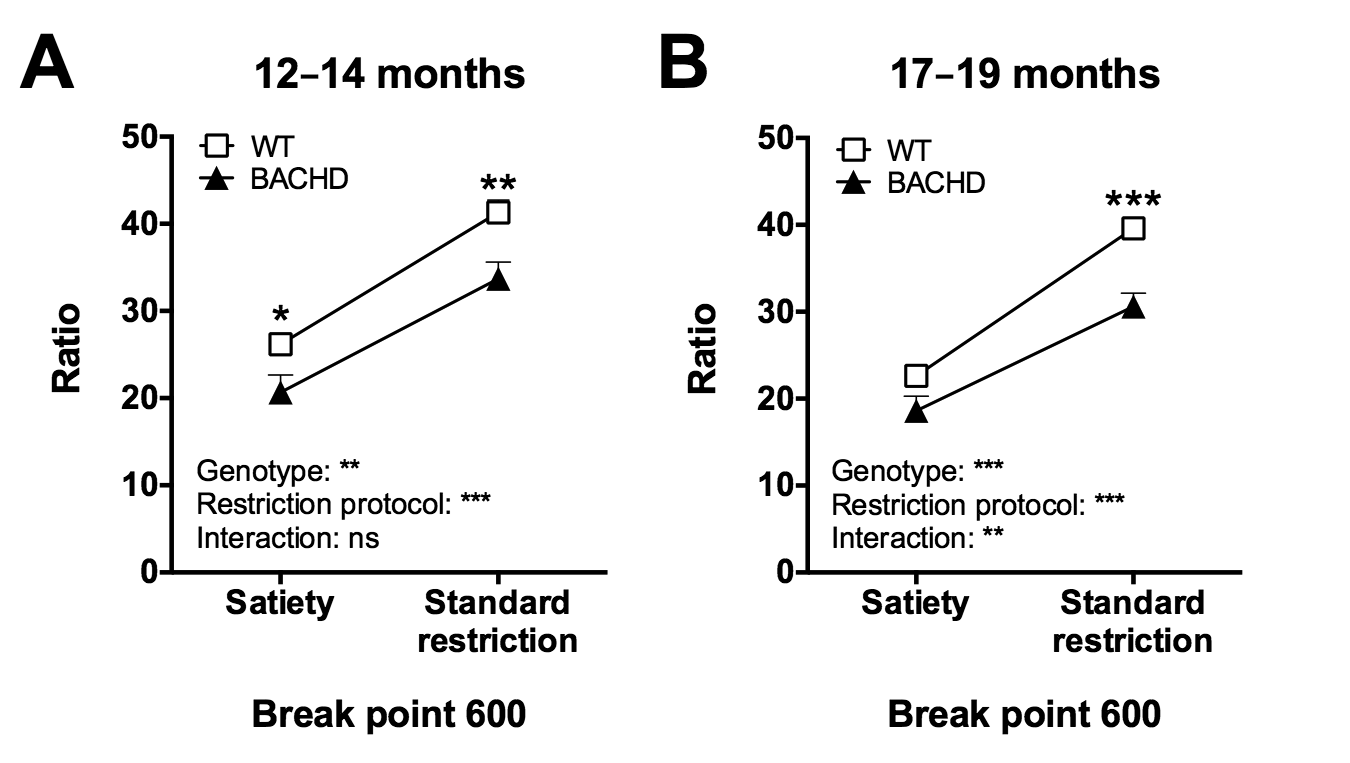

Supplement: S3 Fig — The graphs show comparisons of the number of ratios completed at break point 600 for Group I during their progressive ratio baselines at satiety and the standard food restriction protocol. (A) shows data from the tests performed at 12–14 months of age. (B) shows data from the tests performed at 17–19 months of age. The curves indicate group mean plus standard error, repeated two-way ANOVA results are displayed inside the graphs, and results from post-hoc analysis are shown for individual data points in case significant genotype differences were found. (P < 0.05) *, (P < 0.01) ** and (P < 0.001) ***. (TIFF) [file pone.0173232.s003.tiff]

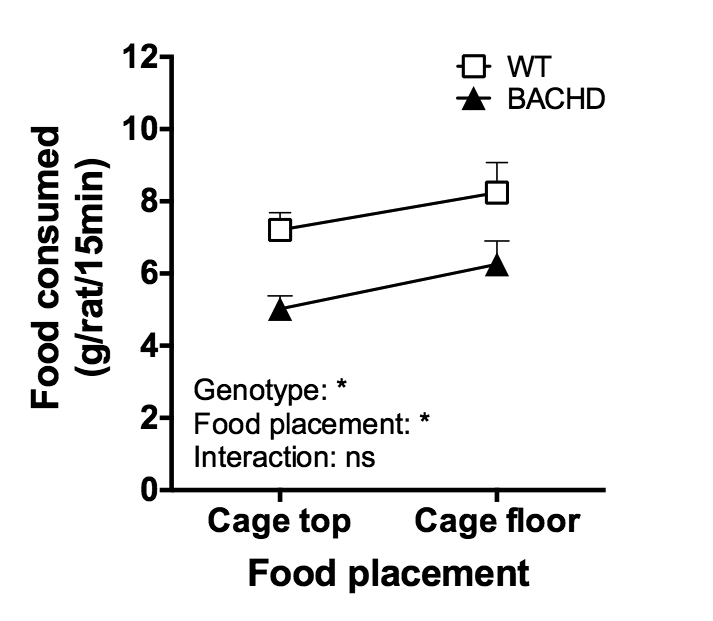

Supplement: S4 Fig — When Group II was maintained on the standard food restriction protocol, one session of the standard food consumption test was run with the food placed inside of the cage (on the cage floor) instead of in the food crib. Data from this session is compared to the performance baseline of the standard food consumption test. The curve indicates group mean plus standard error, repeated two-way ANOVA results are displayed inside the graph. Post-hoc analysis did not reveal significant genotype differences. (P < 0.05) *, (P < 0.01) ** and (P < 0.001) ***. (TIFF) [file pone.0173232.s004.tiff]

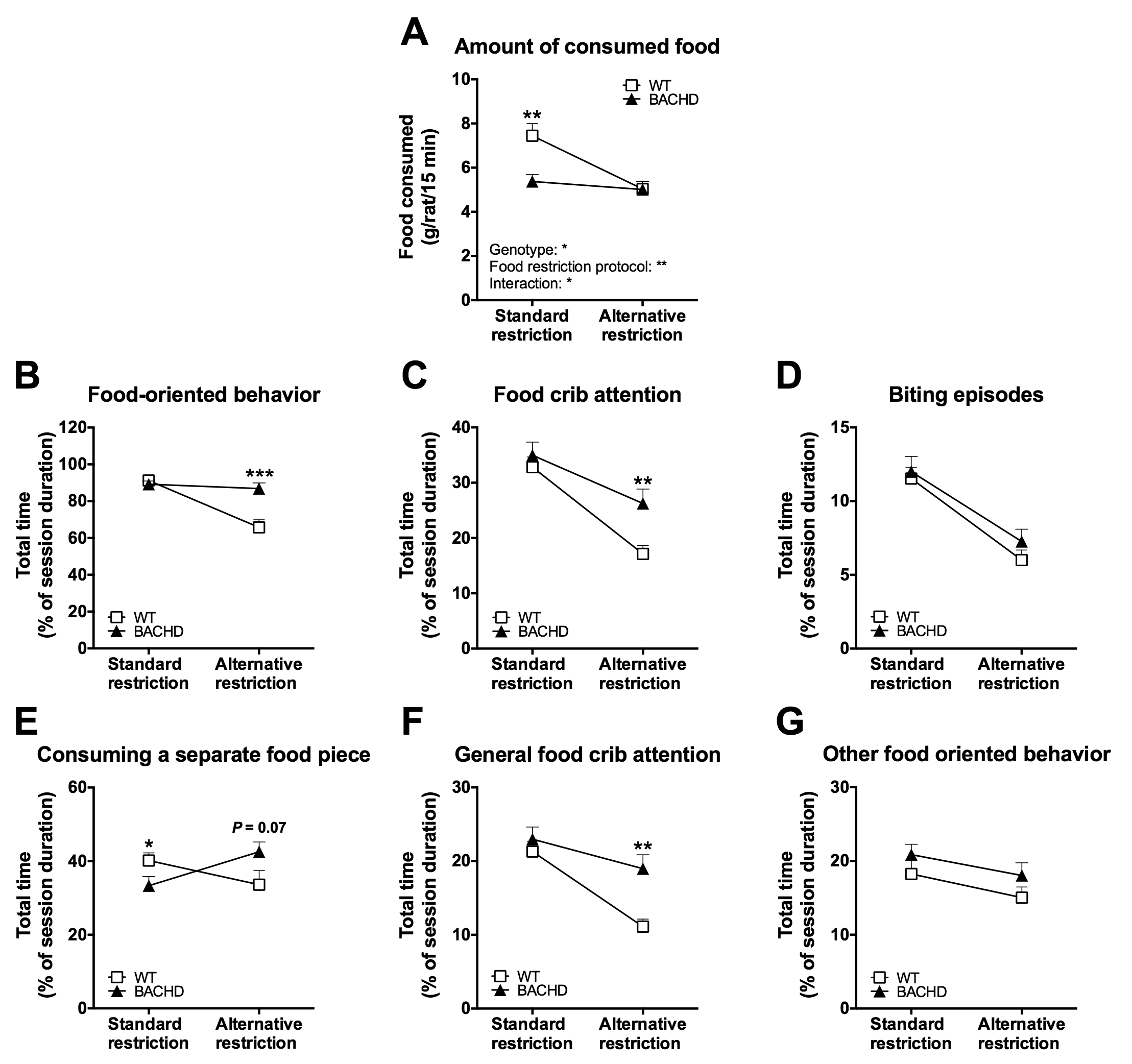

Supplement: S5 Fig — The graphs show the change in Group II’s behavior in the standard food consumption test, when the food restriction protocol was changed from the standard to the alternative approach. Graphs indicate group mean plus standard error. (A) displays results from repeated two-way ANOVA inside the graph and post-hoc analysis at data points where performance between the genotypes differed significantly. (B)–(G) concern the total amount of time spent on the different scored behaviors, and show significant results from t-test or Mann-Whitney test for single comparisons between the genotypes on either restriction protocol (see also Figs 10B and 11B). (P < 0.05) *, (P < 0.01) ** and (P < 0.001) ***. (TIFF) [file pone.0173232.s005.tiff]

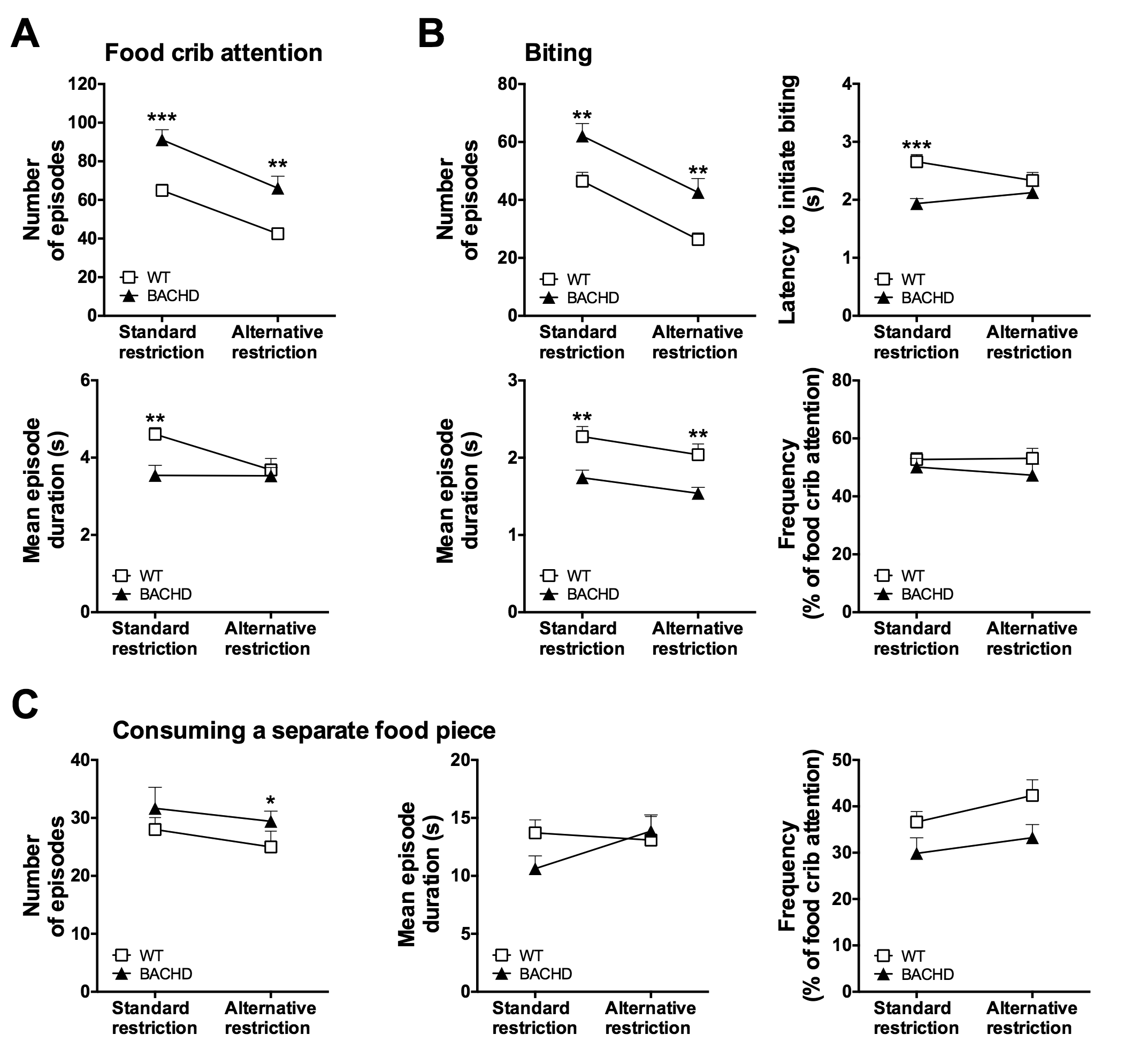

Supplement: S6 Fig — The graphs show the change in Group II’s behavior in the standard food consumption test, when the food restriction protocol was changed from the standard to the alternative approach. Graphs indicate group mean plus standard error. The graphs concern details regarding the number of behavioral episodes, their mean duration, frequency and initiation latency of the different scored behaviors. Significant results from t-test or Mann-Whitney test for single comparisons between the genotypes on either restriction protocol are shown (see also Figs 10C–10E and 11C–11E). (P < 0.05) *, (P < 0.01) ** and (P < 0.001) ***. (TIFF) [file pone.0173232.s006.tiff]

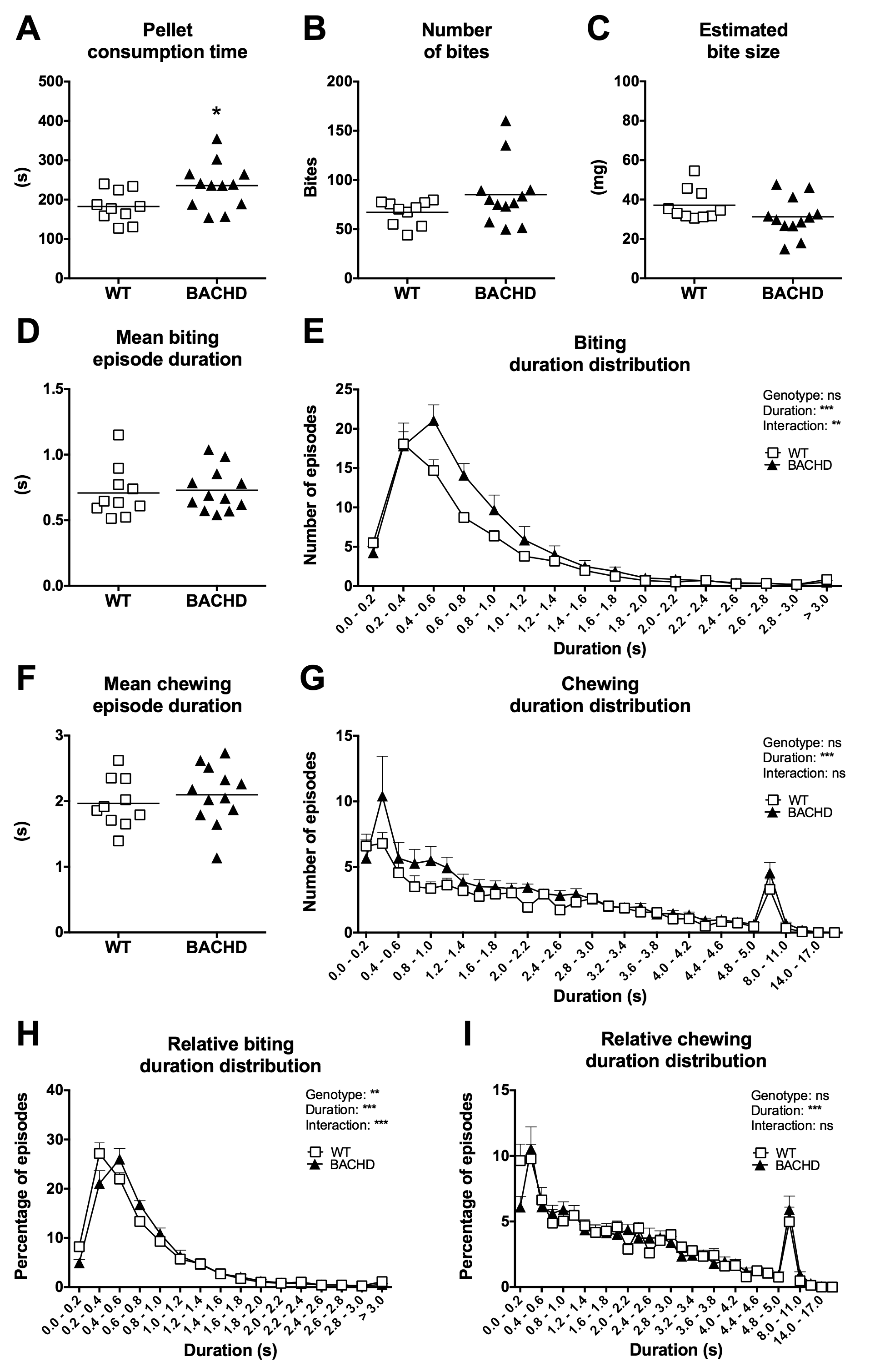

Supplement: S7 Fig — Group II’s mean performance on session 5–7 of the individual food consumption test during the alternative food restriction protocol was subjected to detailed video analysis in order to investigate baseline behavior. (A)–(D) and (F) indicate the performance of individual rats. Significant results from t-test or Mann-Whitney test are shown in case significant genotype differences were found. (E), (G), (H) and (I) show frequency distribution curves for biting and chewing episodes of different durations, indicating group mean plus standard error. The bins used are described in detail in the Material and Methods section. Note that the x-axis in (G) and (I) only labels every other bin. Results from repeated two-way ANOVA are displayed inside the graphs. (P < 0.05) *, (P < 0.01) ** and (P < 0.001) ***. (TIFF) [file pone.0173232.s007.tiff]

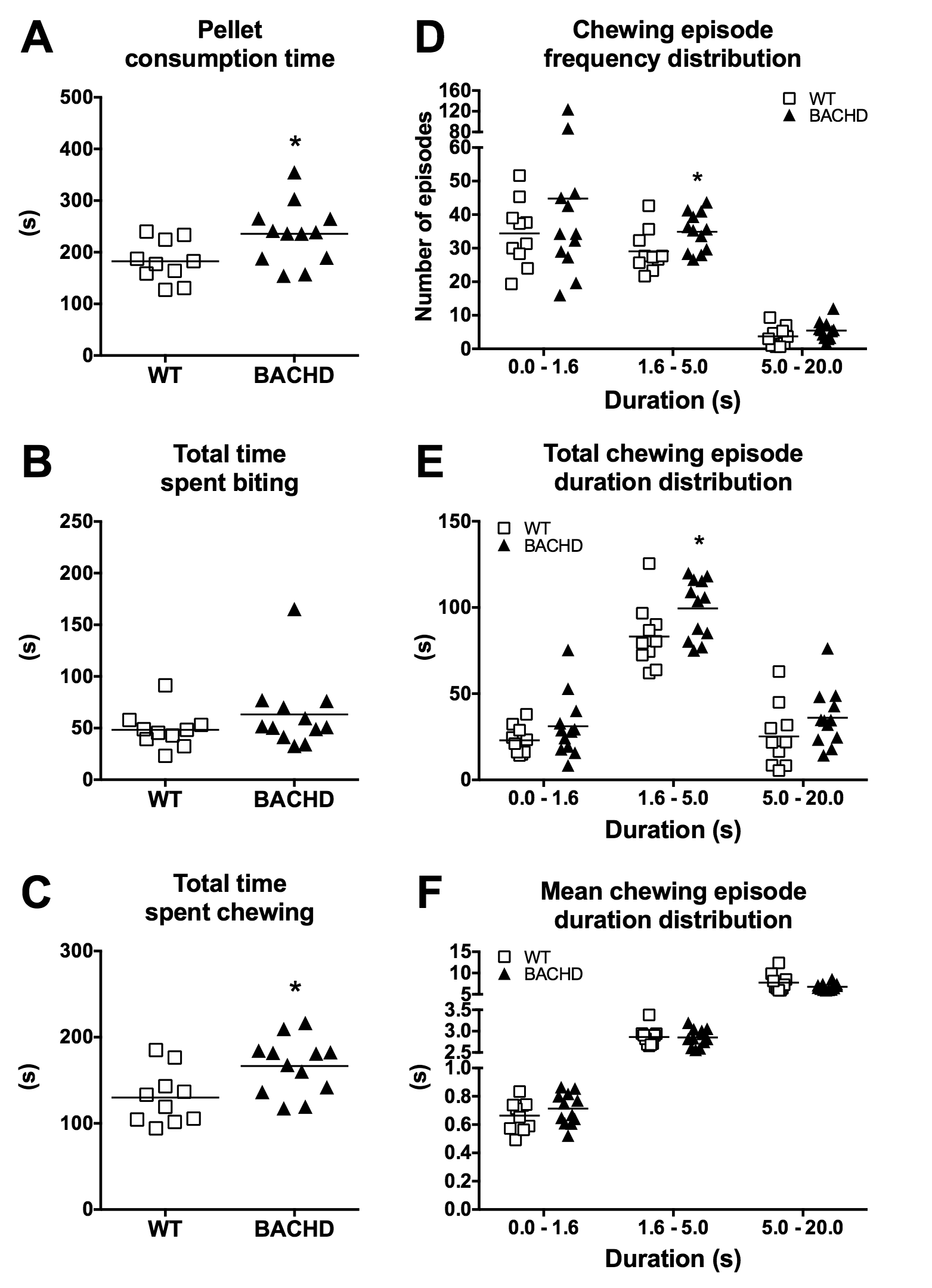

Supplement: S8 Fig — Group II’s performance on session 5–7 of the individual food consumption test during the alternative food restriction protocol was subjected to detailed video analysis in order to investigate baseline behavior. As the initial analysis of these sessions (see S7 Fig) did not clearly reveal the same phenotypes as found in the first session (see Fig 14), additional parameters were analyzed. These particularly concerned the total time spent biting (B) and chewing (C) the food, as well as the frequency distribution of chewing episodes of different durations, using different bins (E) (compare to Fig 14E, 14I and S7E, S7I Fig). Graphs indicate the performance of individual rats and group mean. Significant results from t-test or Mann-Whitney test are shown. (P < 0.05) *, (P < 0.01) ** and (P < 0.001) ***. (TIFF) [file pone.0173232.s008.tiff]

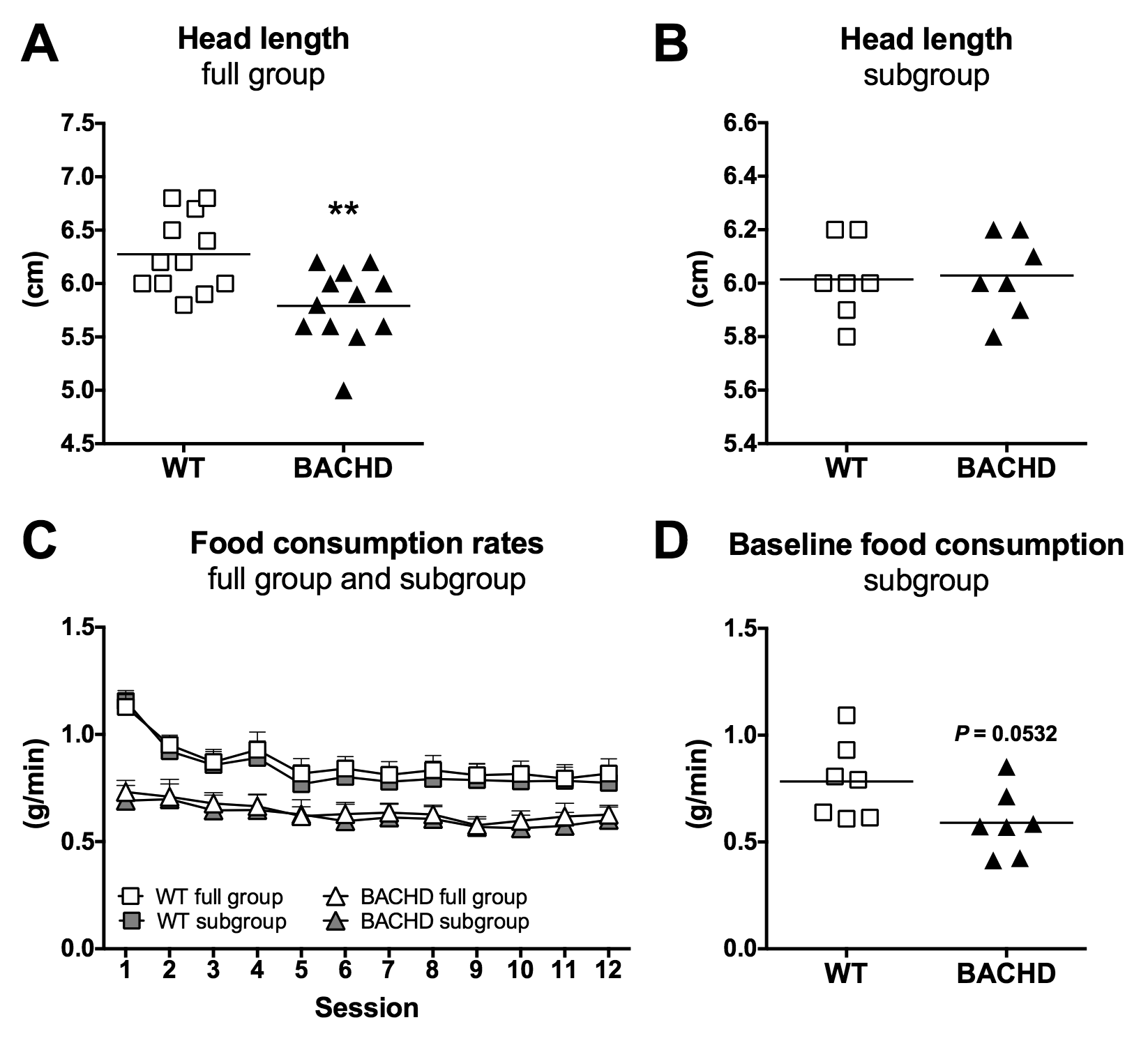

Supplement: S9 Fig — The head size of the rats in Group II was measured at the endpoint of the study, and a brief analysis was made to evaluate if this parameter had any strong influence on the rats' performance. For this, the food consumption of a subgroup of rats with comparable head size was investigated. As noted in previous studies [18], BACHD rats were found to have smaller heads than WT rats (A). (B) displays the comparable head sizes in the subgroup used for further analyses. (C) displays the mean food consumption rates of both the full groups and the subgroups with comparable head sizes (see also Fig 12). (D) shows the mean food consumption rate during baseline performance for the subgroup. (A), (B) and (D) indicate data from individual rats. (C) indicates group mean plus standard error. For (A), (B) and (D), significant results from t-test or Mann-Whitney test are shown. (P < 0.05) *, (P < 0.01) ** and (P < 0.001) ***. (TIFF) [file pone.0173232.s009.tiff]
